# Adenosine mediates the amelioration of social novelty deficits during rhythmic light treatment of 16p11.2 deletion female mice

**DOI:** 10.1038/s41380-024-02596-4

**Published:** 2024-05-13

**Authors:** Jun Ju, Xuanyi Li, Yifan Pan, Jun Du, Xinyi Yang, Siqi Men, Bo Liu, Zhenyu Zhang, Haolin Zhong, Jinyuan Mai, Yizheng Wang, Sheng-Tao Hou

**Affiliations:** 1 Brain Research Centre, Department of Neuroscience, School of Life Sciences, Southern University of Science and Technology, 1088 Xueyuan Blvd, Nanshan District, Shenzhen, 518055 Guangdong PR China; 2 The Brain Science Center, Beijing Institute of Basic Medical Sciences, 100850 Beijing, China; 3 Huashan Hospital, Fudan University, Shanghai, PR China

**Keywords:** Neuroscience, Physiology

## Abstract

Non-invasive brain stimulation therapy for autism spectrum disorder (ASD) has shown beneficial effects. Recently, we and others demonstrated that visual sensory stimulation using rhythmic 40 Hz light flicker effectively improved cognitive deficits in mouse models of Alzheimer’s disease and stroke. However, whether rhythmic visual 40 Hz light flicker stimulation can ameliorate behavioral deficits in ASD remains unknown. Here, we show that 16p11.2 deletion female mice exhibit a strong social novelty deficit, which was ameliorated by treatment with a long-term 40 Hz light stimulation. The elevated power of local-field potential (LFP) in the prefrontal cortex (PFC) of 16p11.2 deletion female mice was also effectively reduced by 40 Hz light treatment. Importantly, the 40 Hz light flicker reversed the excessive excitatory neurotransmission of PFC pyramidal neurons without altering the firing rate and the number of resident PFC neurons. Mechanistically, 40 Hz light flicker evoked adenosine release in the PFC to modulate excessive excitatory neurotransmission of 16p11.2 deletion female mice. Elevated adenosine functioned through its cognate A_1_ receptor (A_1_R) to suppress excessive excitatory neurotransmission and to alleviate social novelty deficits. Indeed, either blocking the A_1_R using a specific antagonist DPCPX or knocking down the A_1_R in the PFC using a shRNA completely ablated the beneficial effects of 40 Hz light flicker. Thus, this study identified adenosine as a novel neurochemical mediator for ameliorating social novelty deficit by reducing excitatory neurotransmission during 40 Hz light flicker treatment. The 40 Hz light stimulation warrants further development as a non-invasive ASD therapeutics.

## Introduction

Autism spectrum disorder (ASD) is a developmental disorder characterized by challenges with social interaction and communication, restricted and repetitive behavior, and intellectual disability [1, 2]. ASD exhibits a higher prevalence among males and has a substantial genetic component. Chromosomal 16p11.2 deletions, in particular, carry a strong genetic risk for ASD [3, 4]. The 16p11.2 deletion mouse carries a deletion of the mouse equivalent of the 16p11.2 region and exhibits a range of behavioral abnormalities that resemble some of the characteristics observed in individuals with the 16p11.2 chromosomal deletion syndrome. Thus, the 16p11.2 deletion mice serve as a valuable tool for studying the genetic and behavioral features associated with the 16p11.2 chromosomal deletion and as a valuable platform for investigating potential therapeutic interventions.

Recently, specific impairment in the ability to integrate information across brain networks has been proposed to contribute to the development of ASD [5]. Impaired power modulation and reduced phase synchronization across multiple frequency bands demonstrate an inability to couple functional neural networks [6]. Specifically, ASD individuals exhibit reduced gamma oscillations during sensory processing [6]. Neuroligin 3 R451C knock-in mice model studies confirmed the presence of gamma oscillation dysfunction in the medial prefrontal cortex (PFC), leading to social deficits [7]. Notably, the combined optogenetic stimulation of the fast-spiking parvalbumin interneurons (PV) at 40 Hz and 8 Hz frequencies effectively rescued the social novelty deficit in these knock-in mice [7]. Currently, it is unknown whether the 16p11.2 deletion mice display gamma oscillation deficits in the PFC. Since synaptic dysfunctions and excitation/inhibition imbalances are common findings in both mouse models, we hypothesized that 40 Hz rhythmic light flicker visual sensory stimulation, known to effectively ameliorate cognitive deficits in animal models of Alzheimer’s disease (AD) [8–10], cerebral ischemia [11], and Parkinson’s disease [12, 13], could entrain and harness regional oscillations in the 16p11.2 deletion brain to have a positive impact on neurological deficits. Therefore, in the present study, we subjected a cohort of female 16p11.2 deletion mice to a long-term 40 Hz light flicker sensory stimulation and investigated the beneficial effects and the underlying mechanisms.

Most published studies on the 16p11.2 deletion mouse model used a mixed population of male and female mice [14–16]. Comprehensive behavioral phenotyping of the 16p11.2 deletion mouse model reported significant differences in neurological behaviors between males and females compared with the control animals [17]. This finding not only confirmed the robust prevalence of ASDs in males relative to females but also indicated a necessity to design experimentation with clear distinctions of the sexes of ASD mice. Information regarding the female 16p11.2 deletion is scarce. Thus, the present investigation focused on the female 16p11.2 deletion mice.

The PFC regulates various behaviors, including social behavior [18, 19]. It is reported that the adenosine A_2A_ receptor (A_2A_R) is involved in the control of PFC-dependent behaviors [20]. Adenosine is a neuro-modulatory molecule that binds to the brain’s four distinct receptor subtypes: A_1_R, A_2A_R, A_2B_R, and A_3_R [21]. The A_1_R and A_3_R have inhibitory effects on excitatory transmission, while the A_2A_R and A_2B_R have excitatory effects [22]. When there is excessive neuronal activity, adenosine can provide feedback inhibition to limit excitability and protect neurons from excitotoxicity [23]. Increased adenosine levels alleviate ASD core symptoms [24]. Furthermore, the activation of both adenosine A_1_ and A_2A_ receptors has been shown to modify the firing rate of neurons in the dorsal striatum and potentially alleviate repetitive behaviors [25, 26]. Indeed, individuals with single-nucleotide polymorphisms in A_2A_R have been found to exhibit heightened autistic symptoms and increased anxiety [27], suggesting that adenosine and its cognate receptors may play a vital role in the development of core symptoms of ASD.

In the present study, we found that female 16p11.2 deletion mice exhibit profound social novelty deficit, which was ameliorated using 40 Hz light flicker treatment for 14 days. Importantly, the 40 Hz light flicker evoked an elevated adenosine expression level, reducing the excessive excitatory transmission in the PFC of the 16p11.2 deletion female mice through an adenosine A_1_R-dependent pathway.

## Materials and Methods

### Animals

Mice with 16p11.2 deletion on a hybrid C57BL/6 N x 129 Sv genetic background were purchased from Jackson Laboratory and bred locally. Female 16p11.2 deletion mice at the age of 2–3 months old were used for the study. Animals were maintained in a condition-controlled room in a pathogen-free SPFII animal facility (23 ± 1 °C, 50 ± 10% humidity). A 12:12 h light/dark cycle (7 a.m. to 7 p.m.) was automatically imposed, and the light intensity was maintained at 15–20 lx during the light period. However, the animal facility room light was at 200 lx during cleaning and experimental operation. Mice were housed in groups of six per individually ventilated cages and given access to food and water *ad libitum*. Experimenters were blinded to animals’ treatments and sample processing throughout the subsequent experimentation and analyses.

### Ethical approval and animal experimentation design

Animal experiment protocols were approved by the Animal Care Committee of the Southern University of Science and Technology (Shenzhen, China). The ARRIVE guideline was followed when designing, performing, and reporting animal experimentation [28]. Mice used in the current study were randomly assigned to each group to maintain total randomization.

According to the ARRIVE reporting guidelines, efforts were made to minimize the number of animals and animal suffering. The inclusion criterion was based on the pre-established identical age and sex of the mice. AEEC Animal Experimentation Sample Size Calculator was used to determine the minimum sample size required to test the study hypothesis [29]. Results indicated the required sample size to achieve 80% power for detecting a 25% difference between two independent means at a significance criterion of α = 0.05 with *n* = 3. A minimum of six mice per group were used in the behavioral studies to achieve meaningful statistical differences. At least three mice per group were used for slice electrophysiology, immunostaining, Golgi staining, fiber photometry recording, microdialysis, and local field potential recording.

### Drug administration

0.2 mg/ml SCH58261 [30] (A_2A_ receptor antagonist, HY-19533, MedChemExpress) and 0.4 mg/mL DPCPX [31] (A_1_ receptor antagonist, HY-100937, MedChemExpress) were dissolved in a solution including 5% DMSO and 95% corn oil. The intragastric administrations of SCH58261 (2 mg/kg) and DPCPX (4 mg/kg) were carried out for 14 days before one-hour 40 Hz visual flicker stimulation. 5 mg/ml adenosine was dissolved in a solution including 2.5% DMSO and 97.5% saline. The intraperitoneal injection of adenosine [32] (100 mg/kg) was carried out for 7 days before behavior tests. During the in vitro electrophysiology recording, 50 mM adenosine (A9251, Sigma) was dissolved in DMSO and diluted to 10 μM in artificial cerebrospinal fluid (aCSF). DPCPX (HY-100937, MedChemExpress) at 1 mM was dissolved in DMSO and diluted in 0.3 μM in aCSF. SCH58261 (HY-19533, MedChemExpress) at 5 mM was dissolved in DMSO and diluted to 0.1 μM in aCSF. The drug administration dosage was determined based on previous studies [22, 33, 34].

### Visual stimulation protocol

The visual stimulation paradigm and methods were essentially as we previously described [11]. The visual stimulation equipment, TangGuangTM, consisted of seven modules, including a tunable frequency signal generator and six LED lamps (36 V). The six LED lamps were connected by parallel circuits and equally distributed around the two transparent cages, which separately housed the control and experimental groups, respectively, at the same time in order for the animals to receive light stimulation simultaneously. The stimulation was conducted once daily at 6–7 pm throughout the experimental duration (up to 14 d). In total, 10 Hz, 40 Hz, 70 Hz light flicker visual stimulation were conducted on mice using the same protocol.

### Open field test

The open-field test was utilized to assess the level of locomotion and anxiety in mice. The method used was as we previously described [35]. Prior to the test, the mice were allowed to acclimate to the testing room for one hour. Each mouse was then placed in the center zone of the open field (40 × 40 x 40 cm). The open field was divided into 16 sections, with the four middle sections (20 cm × 20 cm) designated as the center area. The EthoVision XT software (Noldus Information Technology, Leesburg, USA) was used to record the total distance traveled by the mice and the amount of time spent in the center area during a 10 min period.

### Novel object recognition test

The experiments were conducted in an open field box (40 cm × 40 cm × 40 cm) as we previously described [35]. Before the testing began, the mice were allowed to acclimate to the test room for one hour. During the first stage of testing, two identical plastic toys were placed in the corners of the box, and the mice were given 10 min to explore them. The mice were removed from the box and returned to their home cage. Two hours later, the mice were placed back in the test box, but this time, one of the plastic toys was replaced with a new toy similar in size but different in color and shape. The mice were given 10 min to explore these objects. We measured the time the mouse’s nose tip spent within a statistical range of 5 cm × 5 cm. The EthoVision XT software automatically recorded the exploration time.

### Three-chamber test

The social behavior of mice was studied using a three-chamber apparatus that was divided into three interconnected chambers with transparent plexiglass. The mice were first habituated to the apparatus for 10 min. Sociability was then evaluated during a second 10 min period, during which the test mice could interact with either an empty cage or a genotype, age, and sex-matched stranger mouse (Mouse 1) that was placed in a cage in one of the chambers. Preference for social novelty was then assayed in a third 10 min period by introducing a second stranger mouse (Mouse 2) into the previously empty cage. The time spent interacting with the empty cage, Mouse 1, or Mouse 2, was recorded, and the statistical range of 14 cm × 14 cm was measured using EthoVision XT 10 software. The sociability index (SI) and the social novelty preference index (SNI) were calculated as follows:

$${{{{{\rm{Sociability}}}}}}\; {{{{{\rm{index}}}}}}:\\ = \frac{{interaction\; time\; with\; mouse}1-{interaction\; time\; with\; empty\; cage}}{{interaction\; time\; with\; mouse}1+{interaction\; time\; with\; empty\; cage}}$$

$${{{{{\rm{Social}}}}}}\; {{{{{\rm{novelty}}}}}}\; {{{{{\rm{preference}}}}}}\; {{{{{\rm{index}}}}}}:\\ = \frac{{interaction\; time\; with\; mouse}2-{interaction\; time\; with\; mouse}1}{{interaction\; time\; with\; mouse}2+{interaction\; time\; with\; mouse}1}$$

### Marble-burying test

Mouse cages were placed in the testing room with 5 cm thick bedding and flattened. The mice were transferred to the testing room and were acclimatized for 30 min without being disturbed. The mice were placed in the padded cage for 10 min (without glass marbles) and were taken out. Twenty glass marbles were placed equally in the padded cage in an arrangement of 4 × 5. Then, the mice were put back into the cage and allowed to bury the marbles for 20 min. Those marbles that covered 75% were considered “buried”. The number of buried glass marbles was counted.

### Stereotaxic surgery

The mice were anesthetized with isoflurane and then bilaterally injected with 250 nanoliters of pAAV-U6-shRNA Adora1-CMV-EGFP (concentration at 1.11E + 13 v.g./ml; v.g. viral genome) or pAAV-U6-CMV-EGFP (concentration at 9.67E + 12 v.g./ml) (OBiO Technology, China) into the prefrontal cortex (PFC) at a rate of 0.05 microliters per minute. The injection site was located at AP: +1.98 mm, ML: ±0.3 mm, DV: −2.0 mm from bregma. The needle was left in place for 5 min after injection and then removed. The mice were allowed to recover in their home cage until fully awake. To alleviate pain, meloxicam (1 mg/kg, s.c.) and penicillin (3000 U per mouse, i.p.) were administered once a day for 3 days. The AAV virus was expressed for three weeks to label neurons with EGFP. The nucleotide sequence of the shRNA A_1_ receptor is as follows: 5’-CTCCTTGGGTGTGAATATTGA-3’ [36], and the nonsense nucleotide sequence for the control virus is 5’-CCTAAGGTTAAGTCGCCCTCG-3’.

### Brain slice electrophysiology

The protocol was modified from these studies [35, 37]. Mice were anesthetized with 1% pentobarbital sodium and decapitated. Mouse brain was dissected out and immersed in ice-cold artificial CSF (aCSF) containing (in mM): 30 NaCl, 26 NaHCO_3_, 10 D-glucose, 4.5 KCl, 1.2 NaH_2_PO_4_, 1 MgCl_2_, 194 sucrose, adding 1.5 mL 1 M HCl per 1 L cutting solution and bubbled with 95% O_2_/5% CO_2_. Coronal brain slices (350 μm) were made on a vibratome (VT1120S, Leica Systems). Slices were allowed to recover for 30 min at 34 °C in aCSF containing (in mM): 124 NaCl, 26 NaHCO_3_, 10 D-glucose, 4.5 KCl, 1.2 NaH_2_PO_4_, 1 MgCl_2_, 2 CaCl_2_, adding 10 g sucrose and 1 mL 1 M HCl per 1 L aCSF and bubbled with 95% O_2_/5% CO_2_. After being transferred to a holding chamber at room temperature, the recording started only after at least one hour of recovery. The slices were then placed in a recording chamber (RC26G, Warner Instruments, USA) on the x-y stage of an upright microscope (BX51W; Olympus, Tokyo, Japan) and were perfused with aCSF at a rate of 2 ml/min. All recordings were conducted at room temperature.

To record spontaneous EPSC (sEPSC), sIPSC, and paired-pulse ratio (PPR) in pyramidal neurons, patch recording pipettes were filled with (in mM) 125 CsMeSO_3_, 5 NaCl, 10 HEPES (Na+ salt), 5 QX314, 1.1 EGTA, 4 ATP (Mg2+ salt), 0.3 GTP (Na+ salt). sEPSC was recorded in aCSF with 20 μM bicuculline at −60 mV. sIPSC was recorded in aCSF adding 50 μM D(-)-2-Amino-5-phosphonopentanoic acid (AP5) and 10 μM CNQX at +10 mV. The PPR of the pyramidal neurons in the PFC was recorded to examine the presynaptic glutamate release probability. Electrical stimulation (0.1 ms square pulse) was applied using a glass electrode filled with aCSF and placed within 0.1 mm from the recording site. Throughout the recording, aCSF containing 20 μM bicuculline was perfused. In order to record the PPR, two stimulations with durations of 20 ms, 50 ms, 100 ms, 200 ms, or 500 ms were used. The acquisition frequency was 20.0 kHz, and the filter was set to 2.9 kHz.

To examine neuronal excitability, recording pipettes were filled with (in mM): 128 potassium gluconate, 10 NaCl, 10 HEPES, 0.5 EGTA, 2 MgCl_2_, 4 Na_2_ATP, and 0.4 NaGTP. Current steps ranging from 0 pA to 380 pA with a 20 pA increment and 1 s duration were utilized. The increment was later adjusted to 5 pA to determine the rheobase current, which was defined as the minimum current required to elicit an action potential. The resting membrane potential was measured by injecting a current of 0 pA. The acquisition frequency was set at 20.0 kHz, and the filter was set to 2.9 kHz. sEPSC and sIPSC were analyzed using Mini-analysis (Synaptosoft Inc.). The frequency and amplitude were analyzed. In addition, neuronal spiking traces and PPR traces were imported into Fitmaster software (HEKA Elektronik), and then the number of action potentials and the amplitude of evoked EPSC were measured.

### Immunofluorescence staining

Mice were administered phenobarbital sodium salt (0.1 g/kg) to induce anesthesia. Subsequently, the mice were subjected to transcardial perfusion with ice-cold 0.01 M PBS, followed by 4% (weight/volume) paraformaldehyde (PFA) in 0.01 M PBS. The brains were fixed and cryopreserved in 4% PFA overnight and then dehydrated in 30% sucrose. Coronal sections (30 μm) were prepared and blocked with a solution containing PBS, 0.3% Triton X-100, and 10% goat serum for 1 h at room temperature. The sections were then incubated with primary antibodies overnight at 4 °C. The following primary antibodies were used: anti-PSD95 (rabbit, 1:500, ab18258, Abcam), anti-VGLUT1 (guinea pig, 1:500, 135304, Synaptic Systems), anti-NeuN (mouse, 1:500, ab104224, Abcam), anti-Iba1 (rabbit, 1:500, ab178847, Abcam), anti-CD68 (rat, 1:500, MCA1957, Bio-Rad), anti-PV (rabbit, 1:1000, ab11427, Abcam), anti-GFAP (rabbit, 1:500, ab7260, Abcam) and anti-MAG (mouse, 1:500, ab89780, Abcam). Sections were incubated with appropriate secondary antibodies for 1 h at room temperature. Sections were visualized using a Zeiss LSM980 confocal microscope.

### Analysis of VGLUT1 perisomatic puncta and co-localization with VGLUT1/PSD95

Images were captured by a Zeiss LSM980 confocal microscope with a 100× lens. We collected 3-4 sections from each mouse. For the image analysis of perisomatic puncta, the profile of the cell somata was manually delineated in ImageJ software. To establish the region of interest (ROI), we extended the initial outline by 1 µm from the cell body’s surface. The ROI was then defined as the enclosed area between the two outlines. The overall number of puncta within the ROI was then quantified. The perimeter of the cell somata was calculated. The density of VGLUT1 perisomatic puncta was calculated by dividing the numbers of puncta by perimeter [38, 39]. For VGLUT1/PSD95 analysis, each image was threshold-adjusted using the default auto-threshold and then converted into a binary image. The watershed function was applied to each image to separate overlapping puncta. Co-localization of VGLUT1/PSD95 was analyzed using the ImageJ plugin for co-localization. The binary image of co-localization was used to measure the area and to perform puncta (0.1–1 μm^2^) analysis using a particle analysis tool [40].

### Microglial morphology analysis

For microglial morphology analysis, z-stack images (at 1 μm intervals) were captured by a Zeiss LSM980 confocal microscope with a 63× lens. The z-stack images were projected using the maximum intensity in ImageJ software. Then appropriate plugins (i.e., unsharp mask, despeckle, and close) were consistently used before converting photomicrographs to binary and skeletonized images. The Analyze Skeleton Plugin was then applied to the skeleton image, which tags skeletal features relevant to microglial ramification (i.e., branch length and the number of branches and junctions). We summarized these parameters and normalized all data by the number of microglia cell somas in each image to calculate branch length and the number of branches and junctions per cell [41, 42].

### 3D microglial engulfment analysis

For microglial engulfment assay, z-stack images (at 1 μm intervals) were captured by a Zeiss LSM980 confocal microscope with a 63× lens. Images were captured by randomly selecting microglia with an Iba-1 positive channel without bias. Single microglia, including VGLUT1 signaling, were cropped in Image J and then exported into Imaris 9.0.0 software. First, 3D volume surface renderings of microglia channels were created. The volume and surface area of microglia were calculated. Second, a new channel for VGLUT1 that the microglia have engulfed was created using the mask function. Similarly, the volume of engulfed VGLUT1 was calculated. Engulfment percentage was calculated as the volume of internalized VGLUT1 puncta/volume of microglial cell [43, 44].

### Golgi staining

The FD Rapid GolgiStain^TM^ kit (FD NeuroTechnologies, Ellicott City, MD, USA) was used for the Golgi-cox staining following the manufacturer’s instructions and as previously described [45]. Briefly, the brain was rinsed with double distilled water and then immersed in a 1:1 mixture of FD Impregnation Solution A and B in the dark for three weeks at room temperature. The solution was replaced once after 24 h. After impregnation, we transferred the brains to FD Solution C and stored them in the dark for five days, replacing the solution after 24 h. We mounted individual brains on a specimen disc with an optimum cutting temperature compound to freeze and cut the brains and performed snap freeze and cryosectioning on a Leica CM1950. We cut coronal sections of 200 um thickness and transferred them to agar-treated slides for staining. After drying for 4 days, we rinsed the brain sections twice with double distilled water (dd H_2_O) and stained them in a staining mixture (FD Solution D: FD Solution E: dd H_2_O = 1:1:2 v/v) for 10 min. Then, we rinsed the stained sections again with dd H_2_O twice for 4 min and sequentially dehydrated them in 50%, 75%, 95%, and finally, four times in 100% ethanol, keeping each dehydration immersion step for 4 min. After that, we cleared sections in xylene 3 times for 4 min each rinse and sealed them with resinous mounting medium. The Golgi-stained sections were examined under an optical microscope at 100× magnification. The images were then analyzed using ImageJ software to determine the density per 10 µm of dendritic length.

### Fiber photometry recording and analysis

The mice were anesthetized with isoflurane and then unilaterally injected with 250 nanoliters of rAAV-hSyn-Ado1.0 (Brain Case, China) into the prefrontal cortex (PFC) at a rate of 0.05 microliters per minute. The injection site was located at AP: +1.98 mm, ML: 0.3 mm, DV: −2.0 mm from bregma. The needle was left in place for 5 min after injection and then removed. The mice were allowed to recover in their home cage until fully awake. To alleviate pain, meloxicam (1 mg/kg, s.c.) and penicillin (3000 U per mouse, i.p.) were administered once a day for 3 days. After three weeks, to capture the fluorescence emitted by the adenosine sensor, we affixed an optical fiber to the implanted ferrule via a ceramic sleeve and utilized a commercial fiber photometry system (Thinker Tech Nanjing Biotech CO., Ltd) to record the emissions. The fiber recordings were conducted on freely moving mice during flicker visual stimulation. A MATLAB program developed by Thinkertech was employed to normalize the signal from each continuous experimental trial. The raw signals were initially adjusted to account for photo-bleaching by considering the overall trend before further analysis. To determine the response intensity of adenosine to 40 Hz light flicker visual stimulation, all ΔF/F_0_ values were calculated, a stable baseline was selected, and the SD of the baseline was calculated by selecting the ΔF/F_0_ value of the baseline. When the ΔF/F_0_ value is greater than 3 times the SD and also meets the requirements of the automatic multiscale-based peak detection algorithm [46], it is considered an actual adenosine signal rather than background noise. Finally, the peak and frequency of the adenosine signal were selected in this study to measure the intensity of the adenosine signal.

### Microdialysis and HPLC analysis

An MBR intracerebral guide cannula (MD-2255, BASI) was surgically implanted into the PFC of the mice. The cannula was placed at AP: +1.98 mm, ML: ±0.3 mm, DV: −2.0 mm from bregma. The mice were anesthetized before the experiment, and an MBR-1-5 brain microdialysis probe (MD-2211, BASI) was inserted into the cannula. aCSF (in mM: 124 NaCl, 26 NaHCO_3_, 10 D-glucose, 4.5 KCl, 1.2 NaH_2_PO_4_, 1 MgCl_2_, 2 CaCl_2_, adding 10 g sucrose and 1 mL 1 M HCl per 1 L aCSF and bubbled with 95% O_2_/5% CO_2_) was infused into the microdialysis system at a rate of 2 μl/min using a syringe pump. The dialysates were collected continuously for 60 min under stable lighting conditions. Following one hour of exposure to 40 Hz light flicker, the collection period was extended to 160 min. Dialysates were collected every 20 min while the mice were under anesthesia. The concentration of adenosine in the dialysates was then measured using HPLC analysis.

### Recordings of local field potential (LFP)

#### Implantation of electrodes

The LFP signals in the PFC area were recorded using a method as we previously described [11]. Mice were anesthetized with a mixture of 2.5% isoflurane in N2:O2 (70:30; flow rate 400 ml/min) in an induction chamber. Anesthesia was maintained with 1.5% isoflurane in an N_2_:O_2_ mixture during surgery using an isoflurane vaporizer (Product #R540, RWD Life Science, Shenzhen, China). The animal’s body temperature was maintained at 37.0 ± 0.5 °C using a rectal temperature probe and a heating pad (Product #TCAT-2DF, Harvard Apparatus, USA).

A cranial window at 1.2 mm in diameter with AP: +1.98 mm, ML: ±0.3 mm, DV: −2.0 mm from bregma over the PFC was created using a dental drill (Product #78001, RWD Life Science, Shenzhen, China) guided by stereotaxis (Product # 68861 N, RWD Life Science). A 4-channel microwire array electrode (35 μm, Stablohm 650, California Fine Wire Co., USA) was inserted into PFC and immobilized using kwiksil (Item #KWIK-SIL, Microprobes for Life Science, Gaithersburg, MD, USA). The tetrode was anchored to the skull bone using four skull screws (1.0 mm diameter) and embedded in dental cement. Mice were then moved into a warm (37.0 ± 1 °C) recovery chamber (Product #DW-1, Harvard) for 1 h.

#### LFP recording and analysis

After 4 d recovery from electrode implantation surgery, mice were adapted to the recording position by placing them in a box (50 depth X 50 width X 50 length in cm) for 10 min per day until the first recording session. Before each recording session, the box was cleaned using 75% ethanol. During recording, a helium balloon gently held the implant, allowing the mouse to move freely in the box.

The head stage was connected to the OmniPlex Neural Recording Data Acquisition System (Plexon Inc, Dallas, TX). A camera was set in the front of the box to record the behavioral states of the mouse. An LED lamp was put in front of the box to generate different frequencies of stimulation lights. LFPs were recorded for at least 20 min for each mouse. The LFP signals were sampled at 1000 Hz with a band-pass filter set at 0.5–120 Hz. Raw data were stored for later offline analysis. A total of five mice in each group were used for statistical analysis.

Concurrent with the data recording, we also recorded the behavioral activities of the mice. The specific time intervals were determined by observing the video of the mice’s behavior, and several epochs were selected from the original data, each with the same length of time.

A multitaper fast Fourier transform method was implemented for power spectral analyses using MATLAB (ver 9.11.0, R2021b) software. Data was filtered with a band-pass filter of 0.1 to 100 Hz and a notch filter of 50 Hz. The power spectrum in Fig. 2 was given by a multitaper estimation method using MATLAB using Eq. (1). Given a time series ${X}_{n},n=1,2,\ldots ,N$, the number of the Slepian sequences is $K=2NW-1$. The simplest multitaper estimate of the spectrum is given by

1 $$S(f)=\frac{1}{K}{\sum }_{k=1}^{K}{\left|\frac{1}{N}{\sum }_{n=1}^{N}\exp (2\pi ifn){u}_{n}^{k}{X}_{n}\right|}^{2}$$

where ${u}_{n}^{k},n=1,2,\ldots ,N$ is the kth Slepian sequence.

The Short-Time Fourier Transform (STFT) was applied to reveal the power changes in different frequencies on the time scale. The LFP data was segmented into epochs of the same time interval as described above, and a hamming window with a length of 256, 85% of overlap was used to calculate the spectral amplitude between 1 and 100 Hz through a MATLAB (ver 9.11.0, R2021b) function *stft*.

### Statistical analysis

All data were represented as the Mean ± standard error of the Mean (SEM). All analyses were performed using Prism (V9, GraphPad Software, La Jolla, CA, USA). Data distribution was determined using the Shapiro-Wilk test before applying an appropriate parametric or nonparametric statistical test. If the data from the two groups passed the normality test, an unpaired t-test was used. Otherwise, the Mann-Whitney U test was applied. If the data from several groups passed the normality test, One-way ANOVA with Tukey’s *post hoc* test was used. Otherwise, the Kruskal-Wallis test with Dunn’s *post hoc* test was applied. The firing rate or PPR was analyzed using two-way RM ANOVA with Tukey’s *post hoc* test and Sidak’s *post hoc* test. Statistical details for specific experiments, including the exact n values and what n represents, precision measures, statistical tests used, and the definitions of significance, can be found in figure legends. A *P* < 0.05 was considered statistically significant.

## Results

### Female 16p11.2 deletion mice exhibit social novelty deficit

**Fig. 1:**
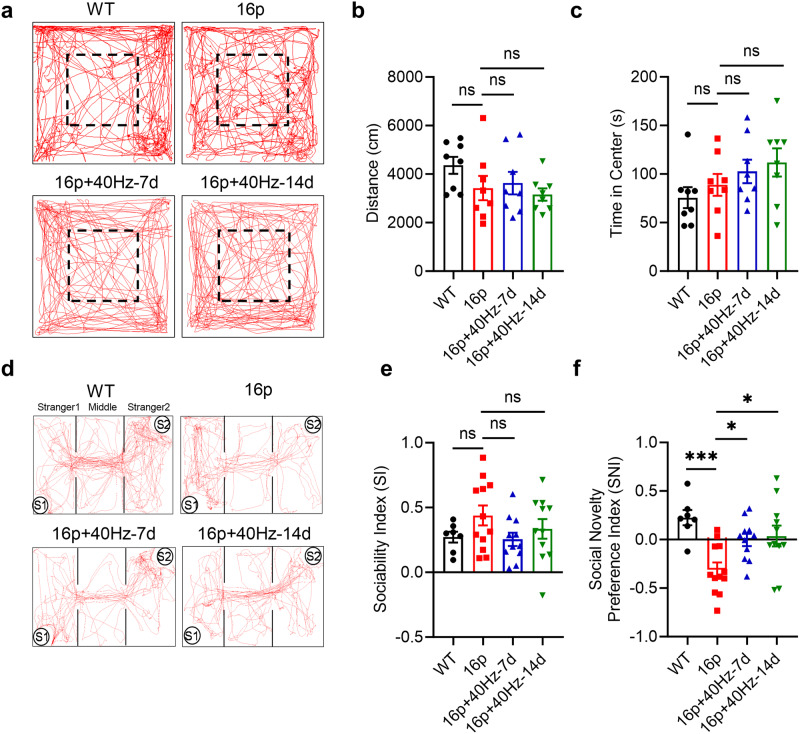
40 Hz light flicker alleviated social novelty deficit in 16p11.2 deletion female mice. **a** The movement trajectory chart of mice in the open field test of all groups. Dashed box: center area. **b** the distance traveled in the open field. **c** time in the center in the open field (Groups: WT, 16p, 16p + 40Hz-7d, 16p + 40Hz-14d; *n* = 8 mice in each group). **d** The trajectory chart of mice in the three-chamber test. S1: familiar mouse, S2: novel mouse. **e** Sociability index in the three-chamber test. **f** Social novelty preference index in three-chamber test (WT: *n* = 7 mice, 16p: *n* = 12 mice, 16p + 40Hz-7d: *n* = 11 mice, 16p + 40Hz-14d: *n* = 11 mice). ns, not significant, *P < 0.05, ***P < 0.001, unpaired t test or one-way ANOVA with Tukey’s *post hoc* test.

We performed several behavioral tests to determine whether the 16p11.2 deletion female mice (labeled as “16p” in all figure panels) have any motor and cognitive dysfunctions compared with the wildtype (WT) female littermates (Fig. 1). In the open field test, the distance traveled and the time spent in the center were not altered in both groups, demonstrating similar locomotion (unpaired t-test, *P* = 0.145) and anxiety levels (unpaired t-test, *P* = 0.408) in the female 16p and WT mice (Fig. 1a–c). However, in the three-chamber test, the female 16p mice showed normal social ability (unpaired t-test, *P* = 0.136) but impaired social novelty ability (unpaired t-test, *P* < 0.001, Fig. 1d–f). In the novel object recognition (NOR) test, the recognition index was also similar, suggesting that the female 16p11.2 deletion mice had a comparable level of cognitive memory to the WT female mice (unpaired t-test, *P* = 0.543, Supplementary Fig. 1a–c). The marble buried test was also performed, showing no difference between the 16p and WT mice (Mann-Whitney U test, *P* = 0.156), indicating no stereotyped behavior in the female 16p mice (Supplementary Fig. 1d, e). In summary, the 16p11.2 deletion female mice had normal locomotion and cognitive memory but impaired social novelty ability.

### Amelioration of social novelty deficit of 16p11.2 deletion female mice by 40 Hz light flicker

The 40 Hz light flicker visual stimulation entrains with oscillations of the visual and higher-order brain cortices, such as the hippocampus, to alleviate cognitive dysfunctions in Alzheimer’s and stroke [11, 47]. However, the effect of a 40 Hz light flicker on neurological deficits in ASD remains unknown. We subjected groups of female 16p11.2 deletion mice to 40 Hz light flicker treatment at 1 h daily for 7 days (40 Hz-7d) and 14 days (40 Hz-14d). On the 8th and 15th days, we performed the open field and three-chamber tests. In the open-field test, the distance traveled and time spent in the open-field center were not altered by the 40 Hz light flicker treatment, suggesting that the light flicker treatment did not affect the locomotion (One-way ANOVA, F_(2, 21)_ = 0.317, *P* = 0.73) and anxiety levels (One-way ANOVA, F_(2, 21)_ = 0.843, *P* = 0.44) of both female 16p11.2 deletion mice (Fig. 1a–c). Moreover, the three-chamber test result also showed that the 40 Hz light flicker was ineffective in altering the social ability of female 16p11.2 deletion mice (One-way ANOVA, F_(2, 31)_ = 1.785, *P* = 0.18, Fig. 1e). In contrast, the 40 Hz light flicker treatment for 7 and 14 days significantly alleviated the social novelty deficit (One-way ANOVA, F_(2, 31)_ = 5.299, *P* = 0.01, Tukey’s *post hoc* test, P_16p vs. 16p+40Hz-7d_ = 0.04, P_16p vs. 16p+40Hz-14d_ = 0.02, Fig. 1d, f). Furthermore, we analyzed the interaction time of female 16p11.2 deletion mice with the familiar (S1) and novel mice (S2). As shown in Supplementary Fig. 1f, 40 Hz stimulation of 16p11.2 deletion female mice significantly reduced the interaction time with the familiar mouse. In comparison, there was a noticeable increase in interaction time with novel mice (S2) in both the 7-day and 14-day treated groups; however, there was no statistical significance. The lack of statistical significance could be attributed to the considerable variability in the response of 16p11.2 deletion female mice to 40 Hz stimulation, as seen in Fig. 1f, which may reflect the diverse spectrum of the disorder.

In addition, the effect of different frequencies of light flicker was measured. Both the 10 Hz and 70 Hz light flicker did not affect locomotion, while 70 Hz light flicker increased the anxiety level in 16p11.2 deletion female mice (One-way ANOVA, F_(2, 19)_ = 4.434, *P* = 0.026, Tukey’s *post hoc* test, P_16p vs. 16p+70Hz-14d_ = 0.025, Supplementary Fig. 2a, b). Moreover, neither 10 Hz nor 70 Hz light flicker could affect sociability and social novelty in 16p11.2 deletion female mice (Supplementary Fig. 2c, d).

To determine the long-term effect of the 40 Hz light flicker treatment, we examined the group of 40 Hz-14d 16p11.2 deletion female mice 2 weeks afterward (16p+2wks after 40 Hz-14d). In the open field test, the distance and time in the center were reduced compared to the 16p11.2 deletion mice (Supplementary Fig. 3a, b), possibly due to the lack of interest in exploring after repeated exposure to the open field. Interestingly, in the social novelty test, the beneficial effect of 40 Hz light flicker treatment disappeared after 2 weeks interval (RM One-way ANOVA, F_(1.768, 15.91)_ = 26.69, *P* < 0.001, Tukey’s *post hoc* test, P_16p vs. 16p+40Hz-14d_ < 0.001, P_16p+40Hz-14d vs. 16p+2 weeks after 40Hz-14d_ < 0.001, Supplementary Fig. 3c, d).

These data showed that the 40 Hz light flicker treatment for 7 and 14 days effectively ameliorated the social novelty disability of the 16p11.2 deletion female mice. However, this beneficial effect requires continuous exposure to the 40 Hz light flicker.

### Suppression of elevated local field potential (LFP) by 40 Hz light flicker in 16p11.2 deletion female mice

**Fig. 2:**
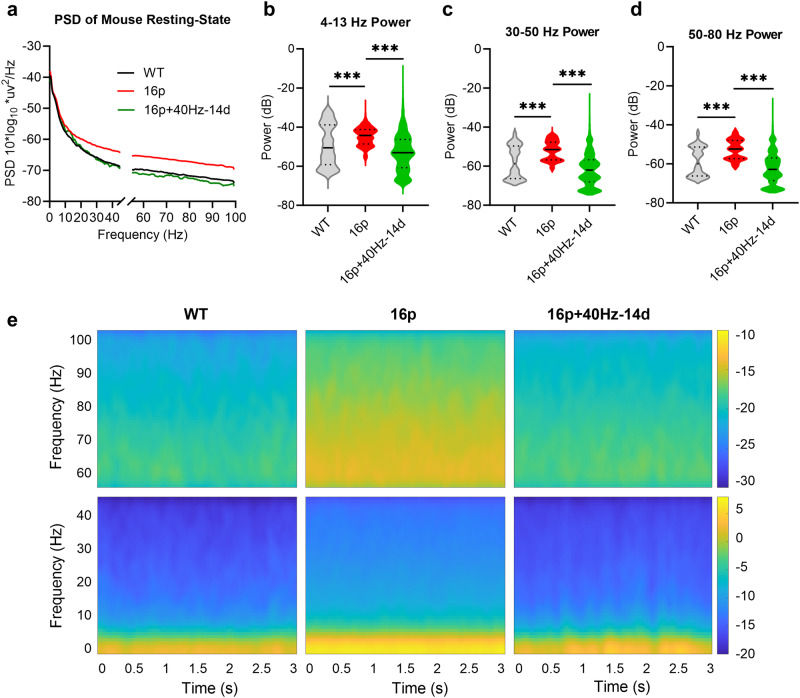
40 Hz light flicker reversed the elevated LFP in 16p11.2 deletion female mice. **a** The power spectral density (PSD) plot of WT, 16p, and 16p + 40Hz-14d groups (*n* = 3 mice in each group). LFP from the PFC region was recorded in free-moving mice in the resting state. All PSDs were multiplied by 10*log_10_ and presented in dB. **b**–**d** Quantification of the PSD of the low-frequency wave (4–13 Hz), low gamma wave (30–50 Hz), and high gamma wave (50–80 Hz), respectively. **e** Time-frequency power representation of the signal using z-scored STFT of the WT, 16p, and 16p + 40Hz-14d groups of mice. ***P < 0.001, Mann-Whitney U test.

To further understand the potential mechanisms of 40 Hz light flicker on ameliorating the social novelty disability of the 16p11.2 deletion female mice, we examined changes in the LFP power in the PFC region. the LFP power wave frequency was divided into the low-frequency waves (4–13 Hz), the low gamma waves (30–50 Hz), and the high gamma waves (50–80 Hz). The LFP power of the untreated 16p11.2 deletion female mice was increased in all frequency bands compared with WT female mice (Fig. 2a–d; Mann-Whitney U test, ***P < 0.001). Interestingly, the LFP power of the 40 Hz-14d 16p11.2 deletion female mice decreased significantly compared to the untreated 16p11.2 deletion female control mice (Fig. 2b–d; Mann-Whitney U test, ***P < 0.001), to a level similar to that of the untreated WT mice (even blow the WT in high gamma regions). Time-frequency spectrogram analysis of digitized signals using z-scored short-time Fourier transform (STFT) of all mice in the groups also showed an increased power of the low and high gamma frequencies in the 16p11.2 deletion female mice (Fig. 2e). These data demonstrated that 40 Hz light flicker modulated the PFC neural network excitation state in the 16p11.2 deletion female mice, which may contribute to the amelioration of the social novelty disability of the 16p11.2 deletion female mice.

### Reduction of excessive excitatory neurotransmission and excitatory synapses by 40 Hz light flicker treatment in 16p11.2 deletion female mice

**Fig. 3:**
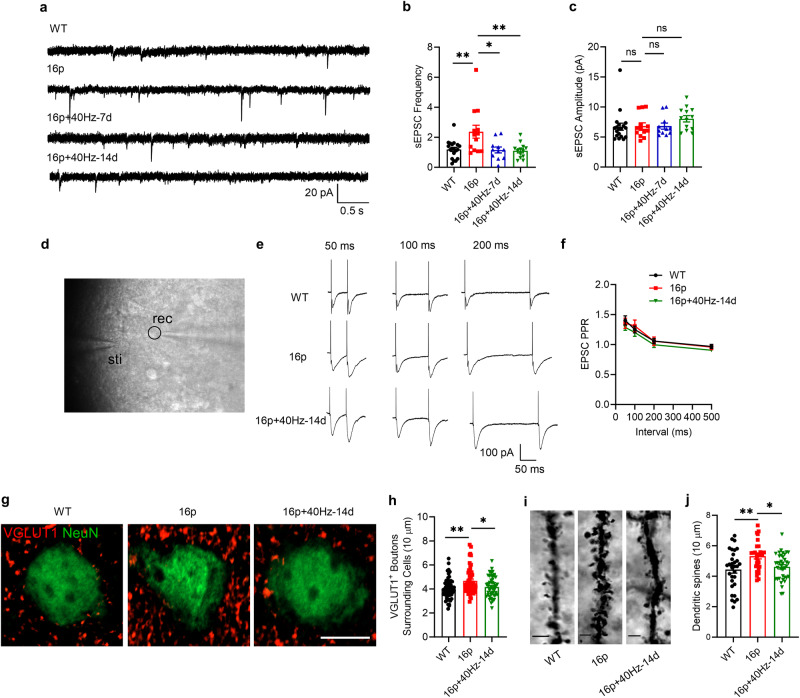
40 Hz light flicker reduced excitatory transmission and excitatory synapses in 16p11.2 deletion female mice. **a** Representative traces of sEPSC recorded in PFC in four groups. **b** Quantification of sEPSC frequency. **c** Quantification of sEPSC amplitude (WT: *n* = 19 cells from 4 mice, 16p: *n* = 13 cells from 3 mice, 16p + 40Hz-7d: n = 11 cells from 3 mice, 16p + 40Hz-14d: *n* = 13 cells from 3 mice). **d** Representative image of paired-pulse stimulation. Sti: stimulating electrode, rec: recording electrode, black circle: recorded neuron. **e** Representative traces of paired-pulse stimulation at the interval of 50 ms, 100 ms, and 200 ms in three groups, scale bar: 100 pA/50 ms. **f** Quantification of EPSC PPR (WT: *n* = 10 cells from 3 mice, 16p: *n* = 9 cells from 3 mice, 16p + 40Hz-14d: *n* = 19 cells from 4 mice). **g** Representative images of VGLUT1 immunostaining in three groups. **h** Quantification of VGLUT1^+^ boutons surrounding cells (WT: *n* = 56 cells from 5 mice, 16p: *n* = 51 cells from 5 mice, 16p + 40Hz-14d: n = 49 cells from 5 mice), scale bar: 10 μm. **i** Representative images of Golgi staining in three groups, scale bar = 3 μm. **j** Quantification of dendritic spines (WT: *n* = 31 dendrites from 3 mice, 16p: *n* = 29 dendrites from 3 mice, 16p + 40Hz-14d: *n* = 38 dendrites from 3 mice). ns, not significant, *P < 0.05, **P < 0.01, unpaired t test, Mann-Whitney U test, Kruskal-Wallis test with Dunn’s *post hoc* test, and one-way ANOVA or two-way RM ANOVA with Tukey’s *post hoc* test.

The spontaneous excitatory postsynaptic current (sEPSC) and spontaneous inhibitory postsynaptic current (sIPSC) were measured, representing excitatory and inhibitory neurotransmission, respectively. We found that the sEPSC frequency, but not the amplitude, was significantly increased in 16p11.2 deletion female mice compared with the WT mice (Fig. 3a–c; sEPSC frequency unpaired t-test, P_WT vs. 16p_ = 0.005; sEPSC amplitude, Mann-Whitney U test, P_WT vs. 16p_ = 0.821). Interestingly, the 40 Hz-7d and 40 Hz-14d significantly reduced the sEPSC frequency (One-way ANOVA, F_(2, 34)_ = 6.138, *P* = 0.005, Tukey’s *post hoc* test, P_16p vs. 16p+40Hz-7d_ = 0.02, P_16p vs. 16p+40Hz-14d_ = 0.01, Fig. 3a, b) without affecting sEPSC amplitude (Kruskal-Wallis test, *P* = 0.22, Fig. 3c). In contrast, neither the frequency nor the amplitude of sIPSC was altered in 16p11.2 deletion female mice compared with the WT mice. Only the 40 Hz-14d, but not the 40 Hz-7d, mice showed induction of sIPSC amplitude on the 14^th^ day (One-way ANOVA, F_(3, 53)_ = 4.759, *P* = 0.0052, Tukey’s *post hoc* test, P_16p vs. 16p+40Hz-14d_ = 0.011, Supplementary Fig. 4). These data showed that long-term 40 Hz light flicker reduced the excitatory neurotransmission in 16p11.2 deletion female mice.

To exclude the possibilities that reduced excitatory neurotransmission was due to an effect on the altered numbers of neurons or the intrinsic differences in excitability of PFC neurons of the 16p11.2 deletion female mice. First, we determined the numbers of NeuN-positive neurons and PV-positive inhibitory interneurons. Results showed that the 40 Hz light flicker did not alter the numbers of these neurons in 16p11.2 deletion female mice (Supplementary Fig. 5). Second, we measured the firing rate of pyramidal neurons in the PFC of the 16p11.2 deletion female mice, which showed a reduced level compared with the WT mice, confirming a previous report [14]. Interestingly, 40 Hz light flicker did not reverse the reduced firing rate of PFC neurons in the 16p11.2 deletion female mice (Two-way RM ANOVA, F_(3, 43)_ = 9.359, *P* < 0.001, Tukey’s *post hoc* test, P_WT vs. 16p_ < 0.001, P_16p vs. 16p+40Hz-7d_ = 0.288, P_16p vs. 16p+40Hz-14d_ = 0.213, Supplementary Fig. 6a–c). Third, the 40 Hz light flicker had no impact on the rheobase current and the resting membrane potential of the PFC pyramidal neurons of the 16p11.2 deletion female mice (rheobase current, unpaired t-test, P_WT vs. 16p_ = 0.003, one-way ANOVA, F_(2, 33)_ = 2.972, *P* = 0.065, P_16p vs. 16p+40Hz-7d_ = 0.053, P_16p vs. 16p+40Hz-14d_ = 0.641, Supplementary Fig. 6d, e).

To further understand the altered neurotransmission, we also measured the PPR, representing the release probability of neurotransmitters. Interestingly, no difference in PPR between the WT and 16p11.2 deletion female mice occurred. Furthermore, the 40 Hz light flicker for 14 days did not affect the PPR (Two-way RM ANOVA, F_(2, 35)_ = 0.972, P = 0.388, Fig. 3d--f).

Could the altered synapses in the 16p11.2 deletion female mice be accounted for the changed excitatory transmission by the rhythmic 40 Hz light flicker? To answer this, we stained the excitatory synapse using a VGLUT1 antibody. We found the number of VGLUT1 positive boutons significantly increased in the 16p11.2 deletion female mice, while 40Hz-14d mice reduced the VGLUT1 positive boutons (unpaired t-test, P_WT vs. 16p_ < 0.001, P_16p vs. 16p+40Hz-14d_ = 0.013, Fig. 3g, h). Furthermore, using Golgi staining, we also found that the dendritic spines were increased in 16p11.2 deletion female mice, which was reduced by 40 Hz light flicker (unpaired t-test, P_WT vs. 16p_ = 0.0042, P_16p vs. 16p+40Hz-14d_ = 0.0025, Fig. 3i, j).

In addition, we co-stained VGLUT1 and PSD95 to label the excitatory synapses. We found that the excitatory synapses were increased in 16p11.2 deletion female mice, which was reduced by 40 Hz light flicker (unpaired t test, P_WT vs. 16p_ = 0.022, P_16p vs. 16p+40Hz-14d_ = 0.002, Supplementary Fig. 7).

Together, these data showed that the 40 Hz light flicker reduced excitatory transmission by reducing the excessive excitatory synapses in the 16p11.2 deletion female mice without affecting the neuronal excitability and density.

### Rhythmic 40 Hz flicker did not enhance the microglia-dependent synaptic pruning

Previous studies using the AD mouse model showed that the 40 Hz light flicker could activate microglia to reduce amyloid plaques [8]. We hypothesized that 40 Hz light flicker might activate microglia to prune excessive excitatory synapses in the 16p11.2 deletion female mice in response to light flicker treatment. We first stained the PFC with Iba1, a microglia marker, to test this hypothesis. We found that the number of microglia did not change in 16p11.2 deletion female mice compared with the WT mice. The 40 Hz for 14 days also did not affect the microglia number (Supplementary Fig. 8a, b). Interestingly, the branch length, branch number, and brunch junction numbers increased in the 16p11.2 deletion female PFC, indicating a more complex microglia morphology than the WT mice. However, 40 Hz light flicker did not affect microglia morphology (Supplementary Fig. 8c–f).

Second, the level of CD68 expression, which is a marker for activated microglia, was determined. Using co-localization studies, we found that both Iba1 intensity and CD68 volume in microglia were reduced in 16p11.2 deletion female mice, while 40 Hz light flicker did not affect Iba1 intensity and CD68 volume in microglia (Supplementary Fig. 8g, h), serving as another piece of evidence. Third, we measured microglia phagocytosis and found that the VGLUT1 volume in microglia was reduced, indicating reduced microglia phagocytosis in 16p11.2 deletion female mice. Rhythmic 40 Hz light flicker also did not affect microglia phagocytosis (Supplementary Fig. 8i, j). Fourth, the level of expression of GFAP and MAG was also determined. The degree of astrogliosis and myelination did not change in 16p11.2 deletion female mice compared with the WT mice, which were neither affected by 40 Hz light flicker (Supplementary Fig. 9). Collectively, these data showed that the 40 Hz light flicker did not activate microglia to engulf and prune excessive excitatory synapses in 16p11.2 deletion female mice.

### Induction of adenosine release by 40 Hz light flicker to suppress A_1_R-dependent excitatory transmission

**Fig. 4:**
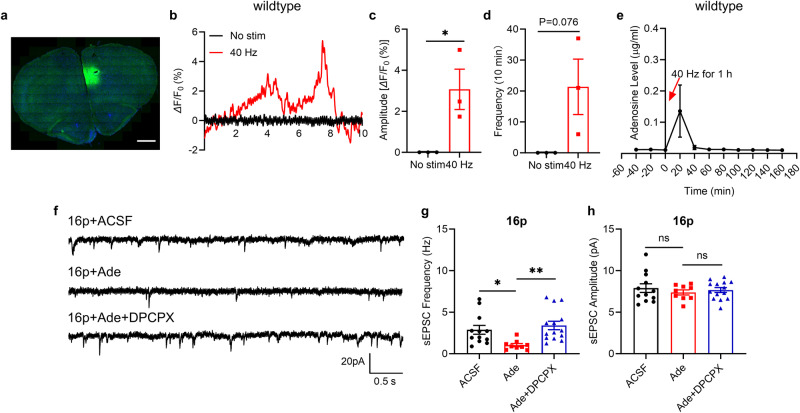
40 Hz light flicker induces the adenosine release. **a** The expression of adenosine sensor in the unilateral PFC (scale bar: 1 mm). **b** Representative trace of adenosine signaling during optical fiber recording. **c** Quantification of the amplitude of adenosine signaling before (10 min) and during 40 Hz light flicker (10 min). **d** Quantification of the frequency of adenosine signaling before and during 40 Hz light flicker (*n* = 3 mice). **e** Quantification of the level of adenosine in PFC of WT female mice before and after one hour of 40 Hz light flicker (*n* = 3 mice). **f** Representative traces of sEPSC in three groups, scale bar: 20 mV/0.5 s. **g** Quantification of sEPSC frequency. **h** Quantification of sEPSC amplitude (aCSF: *n* = 12 cells from 4 mice, Ade (10 mM): *n* = 9 cells from 3 mice, Ade+DPCPX (0.3 mM): *n* = 14 cells from 3 mice). ns, not significant, *P < 0.05, **P < 0.01, unpaired t-test, Kruskal-Wallis test with Dunn’s *post hoc* test, and one-way ANOVA with Tukey’s *post hoc* test.

Adenosine is a potent neuromodulator that strongly suppresses excitatory transmission but has only a minor effect on inhibitory transmission [48, 49]. We hypothesized that 40 Hz light flicker might induce adenosine production to regulate excitatory transmission. We used an adenosine sensor (see Methods) to show the level of adenosine in the PFC of WT female mice and found the peak of fluorescent adenosine signal was very low without light flicker treatment, while 40 Hz light flicker could increase its intensity (unpaired t-test, *P* = 0.036) and frequency (unpaired t-test, *P* = 0.076, Fig. 4a–d). Similarly, 40 Hz light flicker could also increase the fluorescent adenosine signal intensity (unpaired t-test, *P* = 0.074) and frequency (unpaired t-test, *P* = 0.035, Supplementary Fig. 10) in 16p11.2 female mice. This data demonstrated that 16p11.2 female and WT mice release adenosine in response to 40 Hz light flicker treatment.

Moreover, using the microdialysis technique as we previously described [50], we also found that 40 Hz light flicker for 1 hour significantly increased the level of adenosine in the PFC of WT female mice (Fig. 4e).

Importantly, the in-vitro application of adenosine could significantly reduce sEPSC frequency in 16p11.2 deletion female mice, which was blocked by the A_1_R antagonist, DPCPX (Kruskal-Wallis test, *P* < 0.001, Dunn’s *post hoc* test, P_ACSF vs. Ade_ = 0.009, P_Ade vs. Ade+DPCPX_ < 0.001, Fig. 4f–h). In brain slices of 16p11.2 deletion female mice, we also tested the effect of blocking the A_2A_R or A_1_R on excitatory transmission. The A_2A_R antagonist SCH58261 did not affect the sEPSC frequency, but the A_1_R antagonist DPCPX significantly elevated the sEPSC frequency (Kruskal-Wallis test, P < 0.001, Tukey’s *post hoc* test, P_ACSF vs. SCH58261_ = 0.657, P_ACSF vs. DPCPX_ = 0.0035, Supplementary Fig. 11a, b). Neither SCH58261 nor DPCPX affected sEPSC amplitude (Supplementary Fig. 11c).

To confirm whether treatment of adenosine in vivo could alleviate the social novelty deficit, we injected adenosine i.p. into 16p11.2 deletion female mice for 7 days. Adenosine did not affect the locomotion in the open field test (Supplementary Fig. 12a, b). In addition, we observed that adenosine also alleviated the social novelty deficit without affecting sociability in 16p11.2 deletion female mice (unpaired t test, sociability, P_16p+saline vs. 16p+adenosine_ = 0.948, social novelty, P_16p+saline vs. 16p+adenosine_ = 0.024, Supplementary Fig. 12c, d).

### Adenosine A_1_R-dependent reduction of excessive excitatory transmission and alleviation of social novelty deficits

The following experiments were designed to determine whether the rhythmic 40 Hz light flicker evoked adenosine release could contribute to the alleviation of neurological deficits through its cognate receptors. The adenosine receptor consists of excitatory receptors, including A_2A_ and A_2B,_ and inhibitory receptors, including A_1_ and A_3_.

**Fig. 5:**
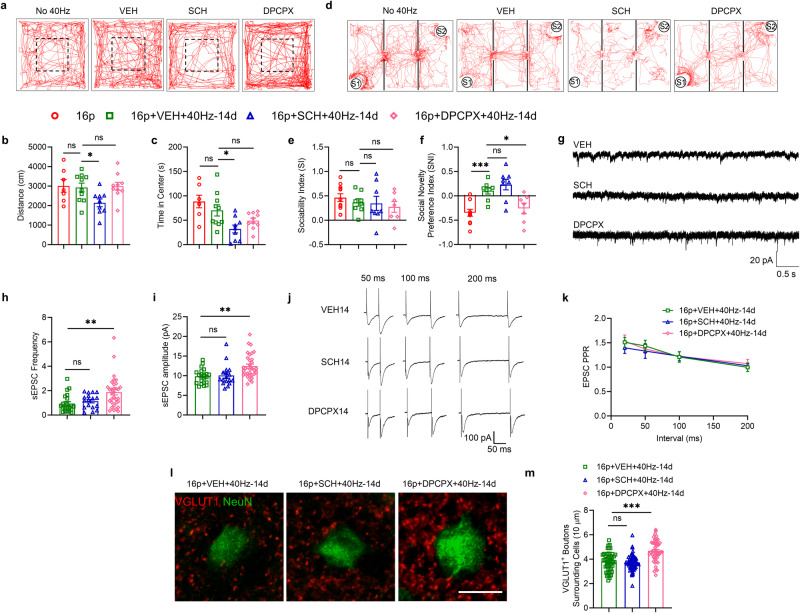
A_1_ receptor antagonist, DPCPX, blocked 40 Hz light flicker’s beneficial effects on social behavior and excitatory transmission. **a** The trajectory chart of mice in the open field in three groups. Dashed box: center area. **b** The distance traveled in the open field. **c** Time in center in open field (16p: *n* = 7, 16p+VEH+40Hz-14d: *n* = 10, 16p+SCH (2 mg/kg)+40Hz-14d: *n* = 9, 16p + DPCPX14 (4 mg/kg)+40Hz-14d: *n* = 10). **d** The trajectory chart of mice in the three-chamber test. S1: familiar mouse, S2: novel mouse. **e** Sociability index in the three-chamber test. **f** Social novelty preference index in three-chamber test (16p: *n* = 11, 16p+VEH+40Hz-14d: *n* = 8, 16p+SCH+40Hz-14d: *n* = 8, 16p+DPCPX+40Hz-14d: *n* = 7). **g** Representative traces of sEPSC recorded in PFC in four groups. **h** Quantification of sEPSC frequency. **i** Quantification of sEPSC amplitude (16p+VEH+40Hz-14d: *n* = 21 cells from 5 mice, 16p+SCH+40Hz-14d: *n* = 17 cells from 4 mice, 16p+DPCPX+40Hz-14d: *n* = 32 cells from 6 mice). **j** Representative traces of paired-pulse stimulation at the interval of 50 ms, 100 ms, and 200 ms in three groups, scale bar: 100 pA/50 ms. **k** Quantification of EPSC PPR (16p+VEH+40Hz-14d: *n* = 13 cells from 3 mice, 16p+SCH+40Hz-14d: *n* = 5 cells from 2 mice, 16p+DPCPX+40Hz-14d: *n* = 11 cells from 3 mice). **l** Representative images of VGLUT1 immunostaining in three groups. **m** Quantification of VGLUT1^+^ boutons surrounding cells (16p+VEH+40Hz-14d: n = 55 cells from 4 mice, 16p+SCH+40Hz-14d: *n* = 58 cells from 5 mice, 16p+DPCPX+40Hz-14d: *n* = 49 cells from 4 mice), scale bar: 10 μm. ns, not significant, *P < 0.05, **P < 0.01, ***P < 0.001, Kruskal-Wallis test with Dunn’s *post hoc* test and one-way ANOVA or two-way RM ANOVA with Tukey’s *post hoc* test.

In the open field test, compared to the control group, the A_2A_R antagonist SCH58261 reduced the locomotion and increased the anxiety level, while in contrast, the A_1_R antagonist DPCPX did not affect locomotion and anxiety (One-way ANOVA, locomotion, F_(2, 26)_ = 4.893, P = 0.016, Tukey’s *post hoc* test, P_16p+VEH+40Hz-14d vs. 16p+SCH+40Hz-14d_ = 0.038, P_16p+VEH+40Hz-14d vs. 16p+DPCPX+40Hz-14d_ = 0.973; anxiety, F_(2, 26)_ = 4.824, *P* = 0.017, Tukey’s *post hoc* test, P_16p+VEH+40Hz-14d vs. 16p+SCH+40Hz-14d_ = 0.013, P_16p+VEH+40Hz-14d vs. 16p+DPCPX+40Hz-14d_ = 0.208, Fig. 5a–c). In the three-chamber test, 40Hz-14d did not affect social ability (unpaired t test, P = 0.404), while both SCH58261 and DPCPX had no effects on social ability (One-way ANOVA, F_(2, 20)_ = 0.172, *P* = 0.844) (Fig. 5e). Interestingly, 40 Hz-14d increased the social novelty preference (unpaired t test, P < 0.001), while DPCPX but not SCH58261 blocked the 40 Hz light flicker’s prosocial effect on social novelty preference (One-way ANOVA, F_(2, 20)_ = 6.776, P = 0.006, Tukey’s *post hoc* test, P_16p+VEH+40Hz-14d vs. 16p+SCH+40Hz-14d_ = 0.614, P_16p+VEH+40Hz-14d vs. 16p+DPCPX+40Hz-14d_ = 0.039, Fig. 5d, f), demonstrating A_1_ receptor’s role in modulating social novelty deficits.

We also measured the sEPSC and found that SCH58261 did not affect the sEPSC frequency and amplitude compared to the control group. In contrast, DPCPX blocked the 40 Hz light flicker’s effect on sEPSC, with significantly increased both the sEPSC frequency and amplitude (Kruskal-Wallis test, frequency, *P* = 0.003, Dunn’s *post hoc* test, P_16p+VEH+40Hz-14d vs. 16p+SCH+40Hz-14d_ = 0.96, P_16p+VEH+40Hz-14d vs. 16p+DPCPX+40Hz-14d_ = 0.003; amplitude, P < 0.001, Dunn’s *post hoc* test, P_16p+VEH+40Hz-14d vs. 16p+SCH+40Hz-14d_ = 0.99, P_16p+VEH+40Hz-14d vs. 16p+DPCPX+40Hz-14d_ = 0.004, Fig. 5g–i). We also measured the PPR. Both SCH58261 and DPCPX did not affect PPR (Two-way RM ANOVA, F_(2, 26)_ = 0.054, *P* = 0.9480, Fig. 5j, k). Furthermore, we stained the excitatory synapse using VGLUT1 and found that only DPCPX blocked the 40 Hz light flicker’s effect on excitatory synapses with increasing VGLUT1^+^ boutons surrounding neurons compared to the control group (One-way ANOVA, F_(2, 159)_ = 25.77, *P* < 0.001, Tukey’s *post hoc* test, P_16p+VEH+40Hz-14d vs. 16p+SCH+40Hz-14d_ = 0.893, P_16p+VEH+40Hz-14d vs. 16p+DPCPX+40Hz-14d_ < 0.001, Fig. 5l, m).

**Fig. 6:**
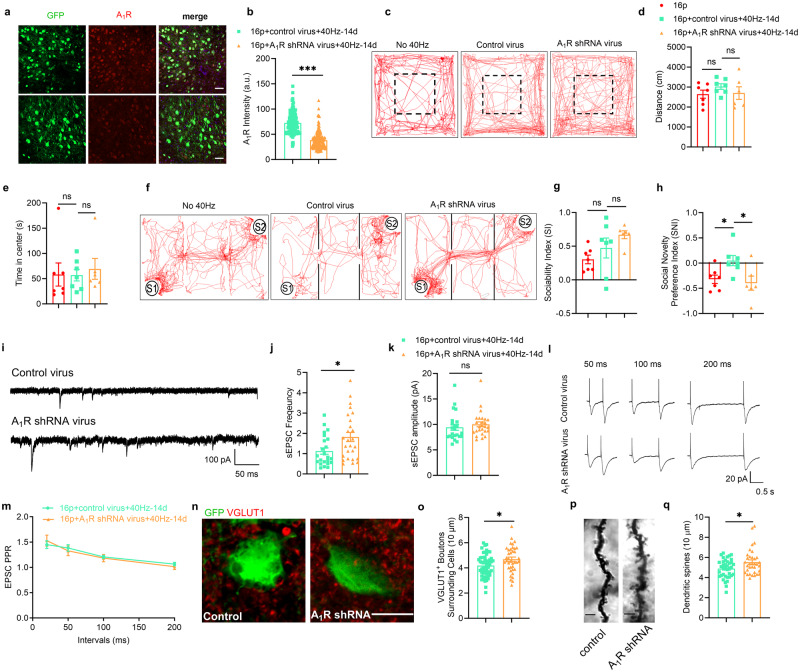
Knockdown of A_1_ receptor blocked 40 Hz light flicker’s beneficial effects on social behavior and excitatory transmission. **a** Representative images of A_1_R immunostaining in two groups, scale bar: 40 μm. **b** Quantification of A_1_R intensity in GFP^+^ cells (16p+control virus+40Hz-14d: *n* = 120 cells from 4 mice, 16p + A_1_ receptor shRNA virus+40Hz-14d: *n* = 150 cells from 5 mice). **c** The trajectory chart of mice in the open field in three groups. Dashed box: center area. **d** The distance traveled in the open field. **e** Time in center in open field (16p: *n* = 7, 16p+control virus+40Hz-14d: *n* = 7, 16p + A_1_ receptor shRNA virus+40Hz-14d: *n* = 6). **f** The trajectory chart of mice in the three-chamber test. S1: familiar mouse, S2: novel mouse. **g** Sociability index in the three-chamber test. **h** Social novelty preference index in three-chamber test (16p: *n* = 7, 16p+control virus+40Hz-14d: *n* = 7, 16p + A_1_ receptor shRNA virus+40H z-14d: *n* = 6). **i** Representative traces of sEPSC recorded in PFC in two groups. **j** Quantification of sEPSC frequency. **k** Quantification of sEPSC amplitude (16p+control virus+40Hz-14d: *n* = 23 cells from 5 mice, 16p + A_1_ receptor shRNA virus+40Hz-14d: *n* = 26 cells from 5 mice). **l** Representative traces of paired-pulse stimulation at the interval of 50 ms, 100 ms, and 200 ms in two groups, scale bar: 100 pA/50 ms. **m** Quantification of EPSC PPR (16p+control virus+40 Hz-14d: *n* = 15 cells from 5 mice, 16p + A_1_ receptor shRNA virus+40Hz-14d: *n* = 15 cells from 5 mice). **n** Representative images of VGLUT1 immunostaining in two groups. **o** Quantification of VGLUT1^+^ boutons surrounding cells (16p+control virus+40Hz-14d: *n* = 51 cells from 4 mice, 16p + A_1_ receptor shRNA virus+40Hz-14d: *n* = 38 cells from 3 mice), scale bar: 10 μm. **p** Representative images of Golgi staining in two groups, scale bar = 3 μm. **q** Quantification of dendritic spines (16p+control virus+40Hz-14d: 38 dendrites from 3 mice, 16p + A_1_ receptor shRNA virus+40Hz-14d: 34 dendrites from 3 mice). ns, not significant, *P < 0.05, ***P < 0.01, unpaired t test, Mann-Whitney U test, and two-way RM ANOVA with Sidak’s *post hoc* test.

To further demonstrate the role of the A_1_ receptor, an adeno-associated virus (AAV) expressing the shRNA to A_1_R (see Methods) was injected into the PFC of 16p11.2 deletion female mice. After 3 weeks of expression, the positive neurons were identified as expressing the reporter eGFP (Fig. 6a). The level of A_1_R expression was significantly reduced in the PFC of 16p11.2 deletion female mice compared to the control group (Mann-Whitney U test, *P* < 0.001, Fig. 6b). We then used these mice to perform several behavioral tests.

In the open field test, compared to the control group, the knockdown of A_1_R had no effects on both locomotion and anxiety (Fig. 6c–e). In the three-chamber test, knockdown of the A_1_R had no effects on social ability (unpaired t test, *P* = 0.27), but blocked the 40 Hz light flicker’s prosocial effect on social novelty preference (unpaired t test, P_16p vs. 16p+control virus+40Hz-14d_ = 0.015, P_16p+control virus+40Hz-14d vs. 16p+A1R shRNA virus+40Hz-14d_ = 0.023, Fig. 6f–h). Compared to the control group, knockdown of A_1_R could block the 40 Hz light flicker’s effect on sEPSC, with significantly increasing the sEPSC frequency (Mann-Whitney U test, *P* = 0.019) without changing sEPSC amplitude (Mann-Whitney U test, *P* = 0.178, Fig. 6i–k). We also measured the PPR and found that the knockdown of A_1_R had no effects on PPR (Two-way RM ANOVA, F_(1, 28)_ = 0.026, *P* = 0.873, Fig. 6l, m). Furthermore, we stained the excitatory synapse using VGLUT1 antibody, and knockdown of A_1_R blocked the 40 Hz light flicker’s effect on excitatory synapses with increasing VGLUT1^+^ boutons surrounding neurons compared to the control group (unpaired t-test, *P* = 0.016, Fig. 6n, o). Finally, using Golgi staining, we found that knockdown of A_1_R increased dendritic spines in 16p11.2 deletion female mice (unpaired t-test, *P* = 0.014, Fig. 6p, q).

Collectively, these data demonstrated that rhythmic 40 Hz light flicker reduced excessive excitatory neurotransmission of 16p11.2 deletion female mice through increasing adenosine release in the PFC. Elevated adenosine, through its cognate A1 receptor, suppresses excitatory transmission, alleviating the social novelty deficits.

## Discussion

In this study, we investigated the effect of rhythmic 40 Hz light flicker stimulation on improving the social novelty deficit of 16p11.2 deletion female mice. The long-term 40 Hz light stimulation for 14 days effectively reduced the elevated power of LFP in the PFC of 16p11.2 deletion female mice and alleviated social novelty deficits. Mechanistically, the 40 Hz light flicker did not alter the firing rate and the number of resident PFC neurons but significantly reversed the excessive excitatory neurotransmission of PFC pyramidal neurons and evoked the production of adenosine. Elevated adenosine levels in the PFC reversed the excessive excitatory transmission through adenosine A_1_R, but not A_2A_R_,_ dependent pathway to alleviate ASD behaviors.

Elucidating biological underpinnings of the robust prevalence of ASDs in males relative to females is of great interest. Recently, a detailed comparison of behavioral phenotypes of 16p11.2 deletion male and female mice showed a clear difference in sex-specific deficits, suggesting gender is an important variable to consider when studying the 16p11.2 deletion mouse model [17]. Indeed, in the present study, we showed that the 16p11.2 deletion female mice exhibited a severe deficit in social novelty ability compared to those of the male mice. Moreover, there is an additional 13% more phenotype occurrence in the female (39.4%) mice than in the male (26.3%) offspring. These findings support the argument that future ASD animal model studies should be designed with clear distinctions of the sexes.

40 Hz was effective in ameliorating the SNI deficits. However, we acknowledge variation in the degree of SNI recovery within the 40 Hz-7d and 40 Hz-14d groups. While some mice exhibited remarkable improvements, others showed a lesser degree of recovery. This heterogeneity in response is an important aspect to consider and highlights the diverse spectrum of disorder variability in this ASD model.

Increased excitation-inhibition (E-I) ratio, observed in several autism mouse models, is an important feature of ASD. Increased excitatory neurotransmission occurs in the pyramidal neurons of 16p11.2 deletion male mice [51] and in human induced pluripotent stem cells-derived dopaminergic neurons carrying 16p11.2 deletion [52]. In the current study, we indeed observed a significant increase in excitatory transmission in the PFC of 16p11.2 deletion female mice, while the inhibitory transmission remained unchanged. Exposure to 40 Hz light flicker for 7 or 14 days reduced the excessive excitatory transmission in these mice, as was reported in the pyramidal neurons of traumatic brain injury mice [53]. Interestingly, the 14-day 40 Hz light flicker treatment also slightly increased inhibitory transmission, suggesting that 40 Hz light flicker may modulate both the excitation and inhibition systems, although a more extended treatment is required to achieve changes in inhibition. Indeed, in an Alzheimer’s disease model, 40 Hz light flicker increases excitatory and inhibitory transmission in the suprachiasmatic nucleus neurons [10]. Our previous findings in a cerebral ischemia mouse model also showed that 40 Hz light flicker increases synaptic glutamate release and enhances LTP [11]. These studies further supported the notion that 40 Hz light flicker alters mouse PFC E-I ratio. In addition, the decreased firing rate of PFC pyramidal neurons was reported in 16p11.2 deletion mice [14, 54], which we also observed in this study. However, 40 Hz light flicker does not increase the firing rate of pyramidal neurons in the PFC of 16p11.2 deletion female mice.

The increased excitatory transmission in 16p11.2 deletion female mice may be attributed to increased synapse density. Although the PPR, indicative of presynaptic release capacity, remained unchanged, immunostaining and Golgi staining revealed increased excitatory synaptic density in the 16p11.2 deletion female mice. Interestingly, a 14-day 40 Hz light flicker treatment reduced the density of excitatory synapses. Previous 40 Hz light flicker studies in AD mouse brains showed activation [8, 55], or inhibition [47] of microglia. However, this study found no change in microglia’s number, morphology, and phagocytosis capacity after 40 Hz light flicker for 14 days. We also did not observe an alteration of microglia in a cerebral ischemia mouse model after 40 Hz light flicker [11]. Thus, suppressing excessive excitation during 40 Hz light flicker may not be due to the microglia’s pruning of excessive synapses.

Adenosine regulates excitatory transmission via excitatory or inhibitory receptors. Adenosine is released from the somatodendritic compartments of neurons through the equilibrate nucleoside transporters [56]. In this study, we found that the 40 Hz light flicker increased the release of adenosine in the PFC area. Furthermore, adenosine inhibited excitatory transmission via A_1_R, but not the A_2A_R, as shown in the in vitro experiments. Our staining experiment showed that the A_2A_R was mainly expressed in the presynaptic, while the A_1_R was expressed in the postsynaptic. Thus, it is highly plausible that 40 Hz-induced adenosine preferentially binds to the A_1_R to modulate the excitatory transmission. Indeed, individuals with ASD exhibit decreased levels of adenosine. Activities that stimulate adenosine production, such as high-intensity physical exercise or elevated body temperature, have been linked to improvements in autism-related behaviors [24]. Furthermore, a specific genetic variation (Asp8Asn) in the adenosine deaminase gene, which results in reduced enzyme activity, has been found to have a significant association with individuals diagnosed with ASD [57, 58]. Thus, increasing adenosine signaling activity might be a novel therapeutic strategy for treating ASD patients. Indeed, we observed that i.p. injection of adenosine for 7 days also alleviated the social novelty deficit without affecting sociability.

It is also worth noting that at postnatal day 50, 16p11.2 deletion mice exhibited altered neurovascular coupling and cerebrovascular reactivity due to endothelial dysfunction [59]. Released adenosine can dilate blood vessels and enhance regional cerebral blood flow [60–62]. Therefore, the 40 Hz light flicker could potentially increase 16p11.2 deletion mice blood flow, supply neurons with the necessary energy and oxygen, and ultimately alleviate the social novelty deficit.

Collectively, these data demonstrated that the 40 Hz light flicker, a non-invasive intervention, modulates PFC function to alleviate 16p11.2 deletion mice’s behavioral deficits. For the first time, we established adenosine as the neurochemical link between 40 Hz light flicker stimulation and improvement in ASD, which warrants further studies to establish the adenosine signal transduction pathway as an effective therapeutic intervention for ASD.

A limitation of this mouse model is that it represents only 5.5‰ of the cases of autism caused by genetic factors. The effects of 40 Hz light flickers on other more common murine models of autism, like SETD5, mutated in 1% of the cases of autism [63], should be tested and compared with the current data to establish 40 Hz treatment as a clinically relevant therapy.

## Supplementary information

Supplementary figures and figure legends

## Data Availability

All primary data are archived at the Southern University of Science and Technology and available upon request.
