# Supplementary material for: Adenosine mediates the amelioration of social novelty deficits during rhythmic light treatment of 16p11.2 deletion female mice
Source: Mol Psychiatry. 2024 May 13;29(11):3381–94. doi: 10.1038/s41380-024-02596-4 (PMC11541200; doi:10.1038/s41380-024-02596-4)
Supplement: Supplementary file 1 — Supplementary figures and figure legends [file 41380_2024_2596_MOESM1_ESM.docx]

**Adenosine mediates the amelioration of social novelty deficits during rhythmic light treatment of 16p11.2 deletion female mice**

Jun Ju^1,#^, Xuanyi Li^1,#^, Yifan Pan^1^, Jun Du^2^, Xinyi Yang^1^, Siqi Men^1^, Bo Liu^1^, Zhenyu Zhang^1^, Haolin Zhong^1^, Jinyuan Mai^1^, Yizheng Wang^3^, Sheng-Tao Hou^1,*^

1 Brain Research Centre, Department of Biology, School of Life Sciences, Southern University of Science and Technology, 1088 Xueyuan Blvd, Nanshan District, Shenzhen, 518055, Guangdong, P. R. China.

2 The Brain Science Center, Beijing Institute of Basic Medical Sciences, 100850 Beijing, China.

3 Huashan Hospital, Fudan University, Shanghai, P. R. China.

^#^ These authors contributed equally: Jun Ju, Xuanyi Li

* Corresponding author:

Prof. Sheng-Tao Hou, email: hou.st@sustech.edu.cn

**Supplementary Figures and Figure Legends**

**
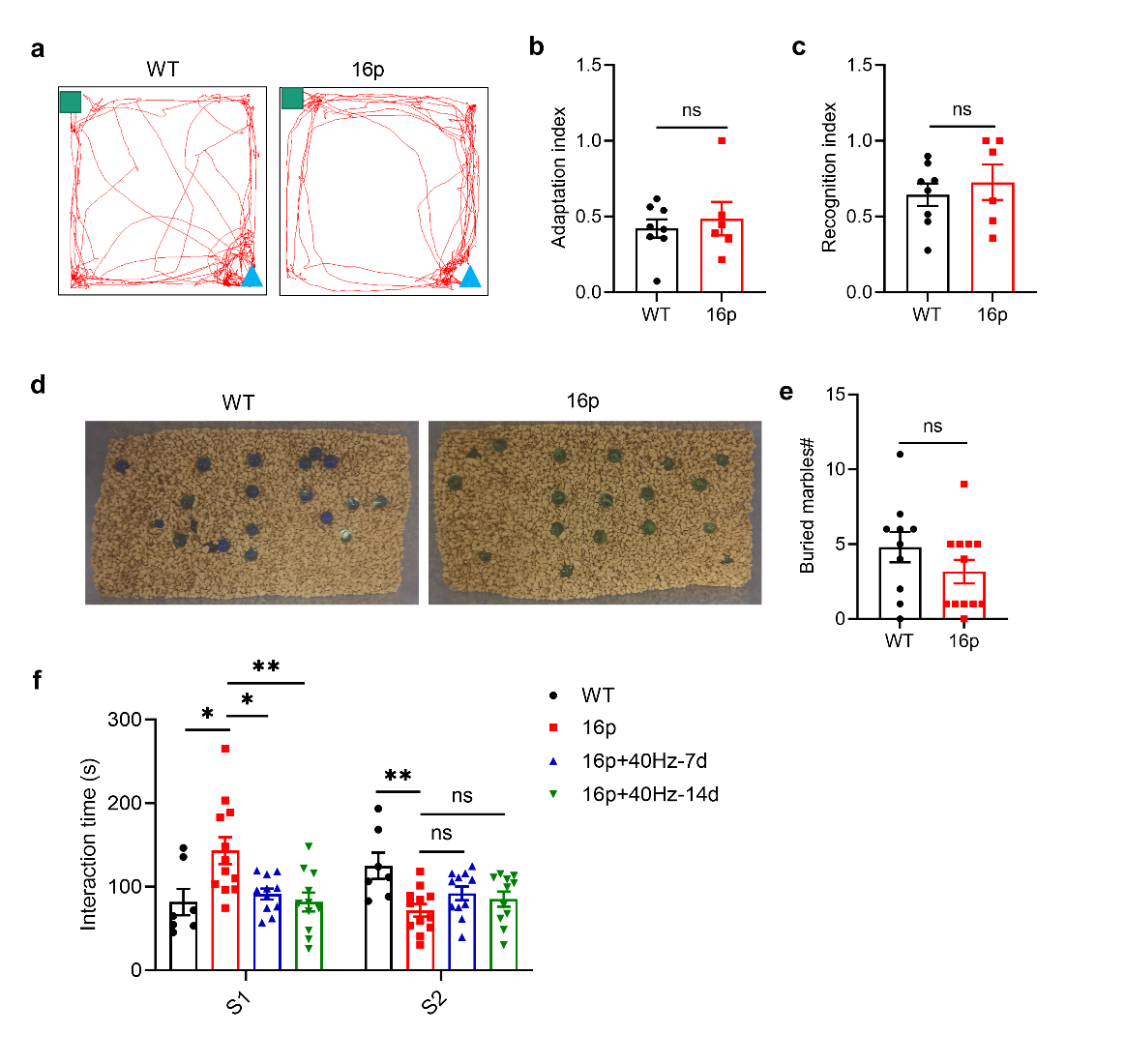
**

**Supplementary Fig. 1 16p11.2 deletion female mice showed normal cognitive memory and stereotyped behavior**

**a** The trajectory chart of mice in novel object recognition test (NOR). Green square: familiar object, blue triangle: novel object. **b** Adaption index in the NOR. **c** Recognition index in the NOR (WT: n = 8 mice, 16p: n = 6 mice). **d** The representative images of buried marbles. **e** The number of buried marbles (WT: n = 10 mice, 16p: n = 12 mice). **f** The interaction time of mice with familiar mouse or novel mouse (WT: n = 7 mice, 16p: n = 12 mice, 16p+40Hz-7d: n = 11 mice, 16p+40Hz-14d: n = 11 mice). ns, not significant, *P < 0.05, **P < 0.01, unpaired t test, Mann-Whitney U test, and one-way RM ANOVA with Tukey's *post hoc test*.

**
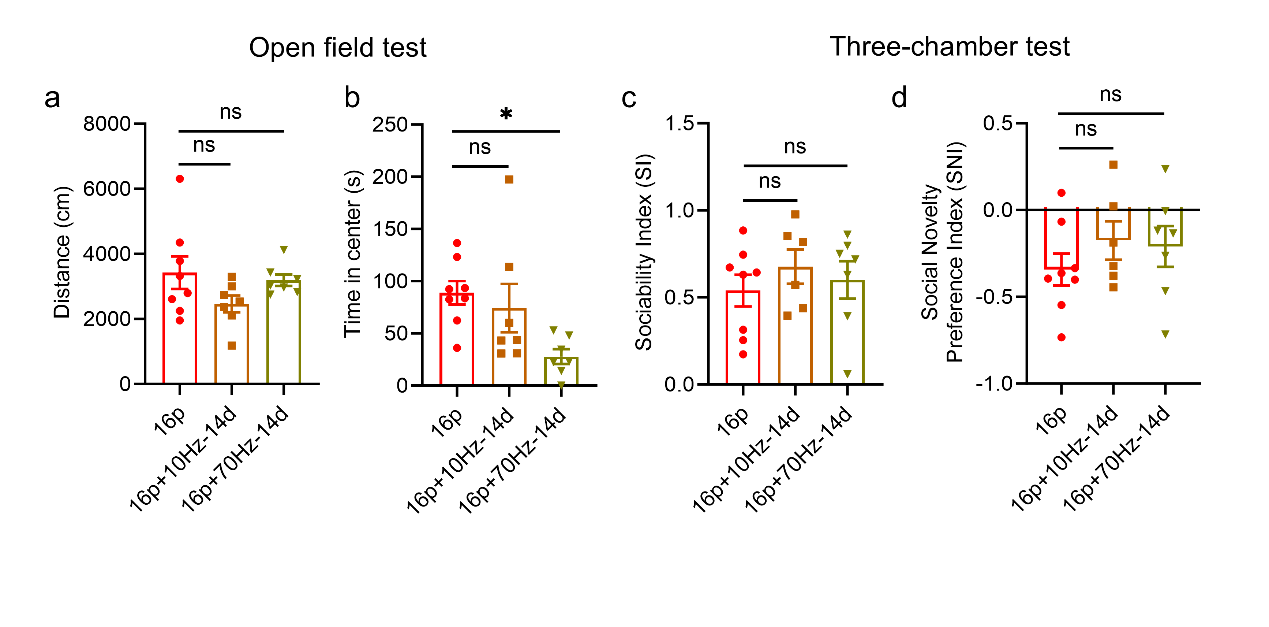
**

**Supplementary Fig. 2 10 Hz and 70 Hz light flicker did not alleviate social novelty deficit in 16p11.2 deletion female mice**

**a, b** The distance traveled in the open field and the time in center in open field (16p: n = 8 mice, 16p+10Hz-14d: n = 7 mice, 16p+70Hz-14d: n = 7 mice), respectively. **c** Sociability index in the three-chamber test. **d** Social novelty preference index in three-chamber test (16p: n = 8 mice, 16p+10Hz-14d: n = 6 mice, 16p+70Hz-14d: n = 7 mice). ns, not significant, *P < 0.05, one-way ANOVA with Tukey's *post hoc* test.

**
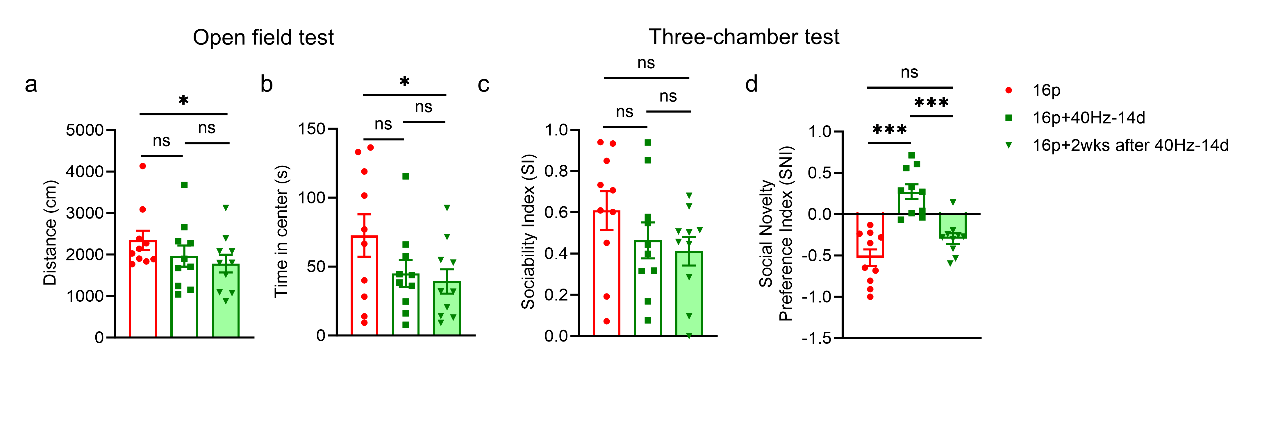
**

**Supplementary Fig. 3 40 Hz for 14 days did not have a long-term therapeutic effect**

**a** The distance traveled in the open field, and **b** Time in the center in the open field (16p: n = 10 mice, 16p+40Hz-14d: n = 10 mice, 16p+2wks after 40Hz-14d: n = 10 mice). **c** Sociability index in the three-chamber test, and **d** Social novelty preference index in the three-chamber test (16p: n = 10 mice, 16p+40Hz-14d: n = 10 mice, 16p+2wks after 40Hz-14d: n = 10 mice). ns, not significant, *P < 0.05, ***P < 0.001, one-way RM ANOVA with Tukey's *post hoc* test.

**
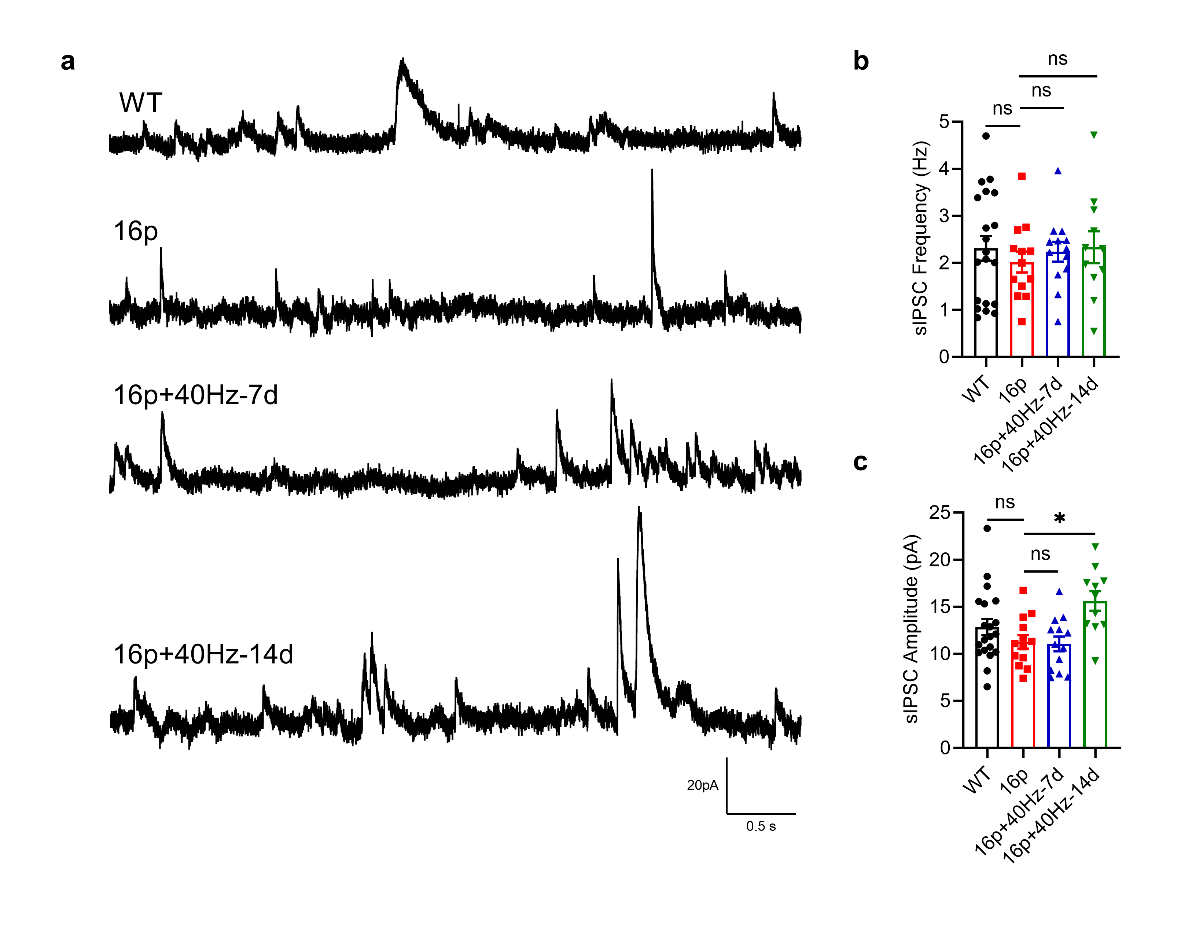
**

**Supplementary Fig. 4 40 Hz light flicker slightly increased GABAergic transmission**

**a** Representative traces of sIPSC recorded in PFC in four groups, scale bar: 20 mV/0.5 s. **b** Quantification of sIPSC frequency. **c** Quantification of sIPSC amplitude (WT: n = 20 cells from 4 mice, 16p: n = 13 cells from 3 mice, 16p+40Hz-7d: n = 13 cells from 3 mice, 16p+40Hz-14d: n = 11 cells from 3 mice). ns, not significant, *P < 0.05, one-way ANOVA with Tukey's *post hoc* test.


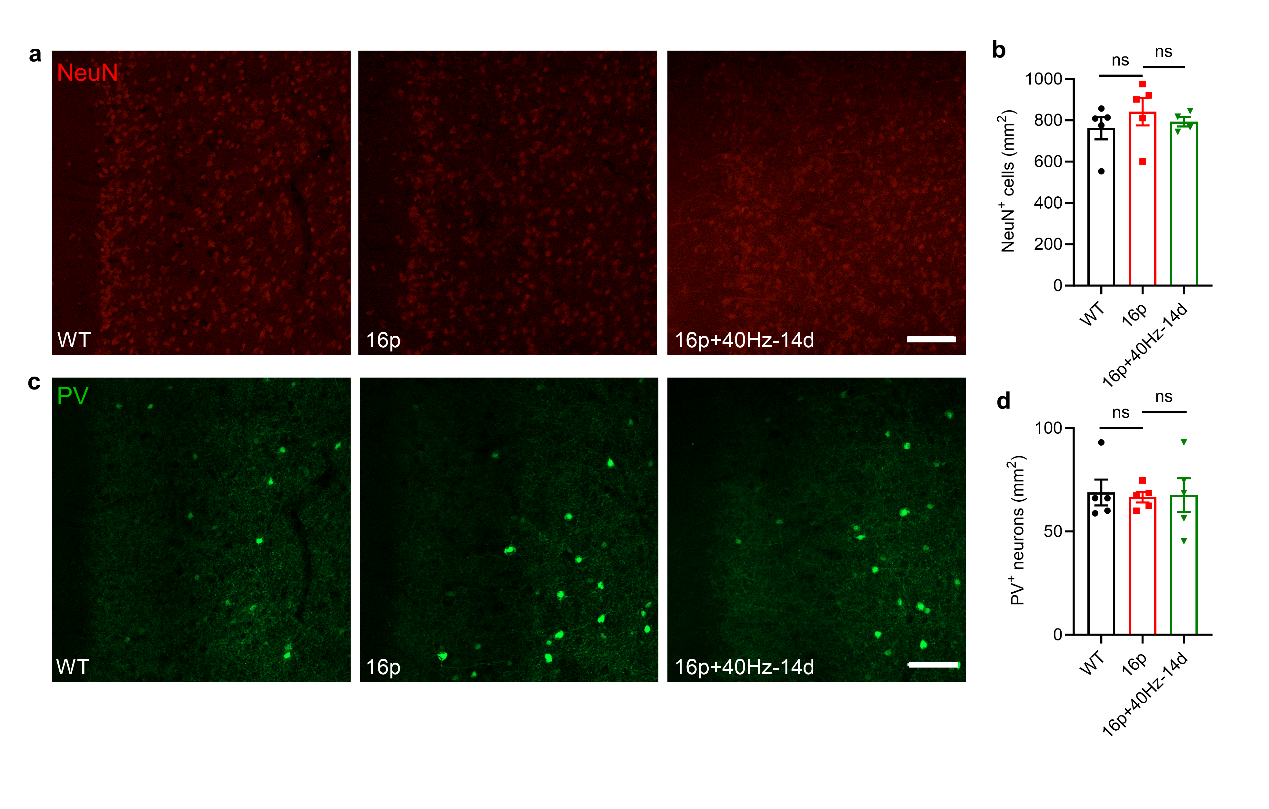


**Supplementary Fig. 5 40Hz flicker did not affect the number of mature neurons or PV+ neurons**

**a** Representative image of neurons in three groups, scale bar: 100 μm. **b** Quantification of NeuN^+^ cells density (WT: n = 5 mice, 16p: n = 5 mice, 16p+40Hz-14d: n = 4 mice). **c** Representative image of PV neurons in three groups, scale bar: 100 μm. **d** Quantification of PV^+^ neurons density (WT: n = 5 mice, 16p: n = 5 mice, 16p+40Hz-14d: n = 5 mice). ns, not significant, one-way ANOVA with Tukey's *post hoc* test.

**
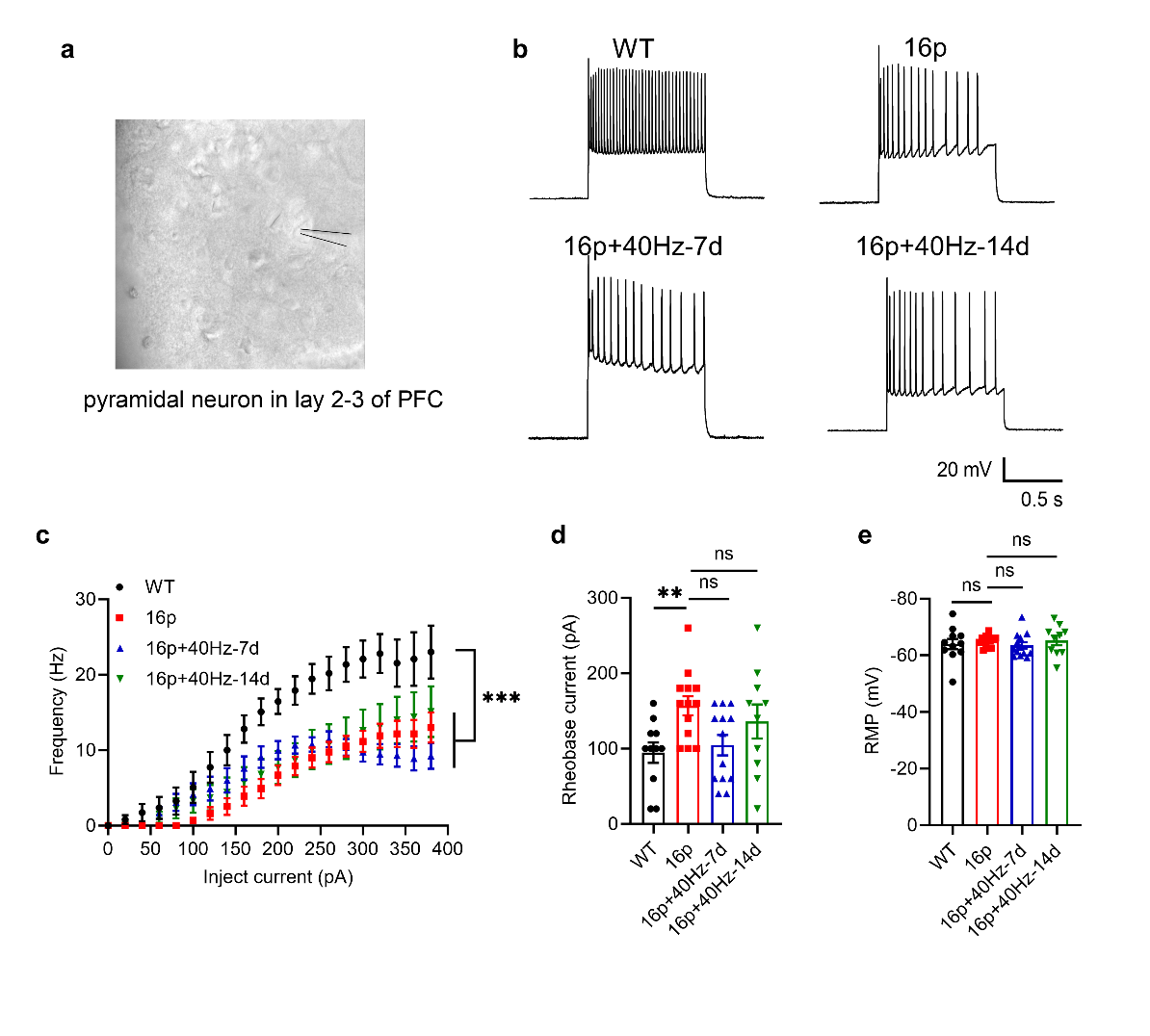
**

**Supplementary Fig. 6 40 Hz light flicker did not elevate the reduced firing rate in 16p11.2 deletion female mice**

**a** Representative image of slice recording in PFC. **b** Representative traces of firing rate of pyramidal neurons in four groups, scale bar: 20 mV/0.5 s. **c** Quantification of firing rate in pyramidal neurons. **d** Quantification of rheobase current. **e** Quantification of resting membrane potential (WT: n = 11 cells from 3 mice, 16p: n = 13 cells from 4 mice, 16p+40Hz-7d: n = 13 cells from 3 mice, 16p+40Hz-14d: n = 10 cells from 3 mice). ns, not significant, **P < 0.01, ***P < 0.001, unpaired t test and two-way RM ANOVA with Tukey's *post hoc* test.


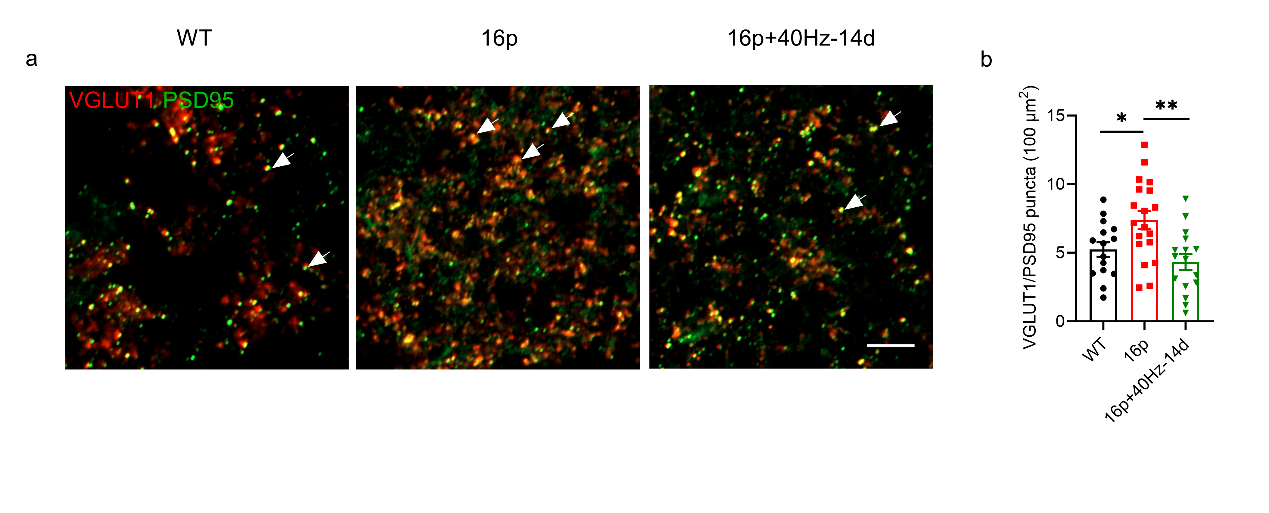


**Supplementary Fig. 7 40 Hz light flicker reduced the excitatory synapses in 16p11.2 deletion female mice**

**a** Representative images of VGLUT1/PSD95 immunostaining in three groups, scale bar: 5 μm. **b** Quantification of excitatory synapse density (WT: n = 15 fields from 5 mice, 16p: n = 19 fields from 5 mice, 16p+40Hz-14d: n = 16 fields from 5 mice). *P < 0.05, **P < 0.01, unpaired t test.


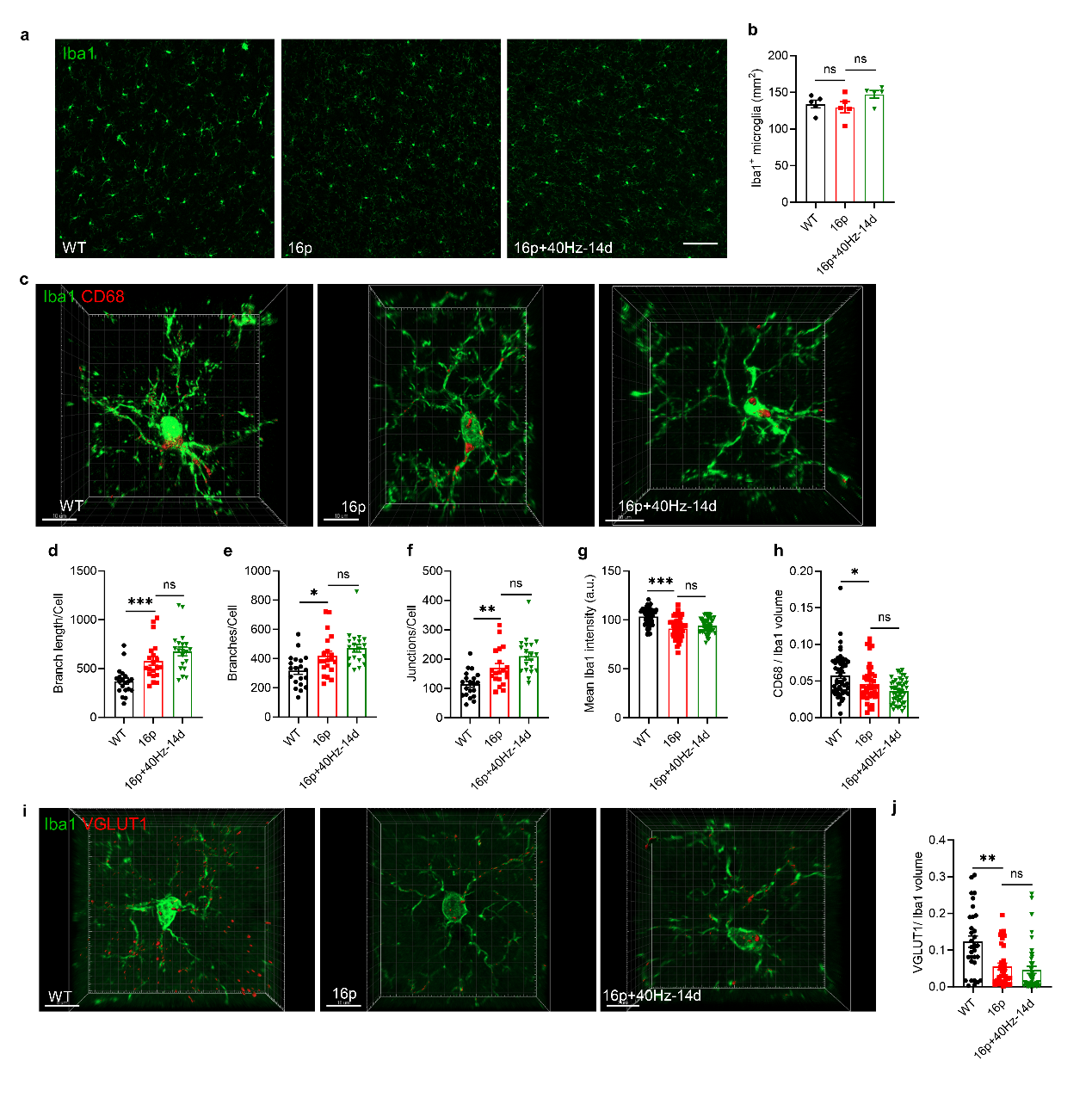


**Supplementary Fig. 8 40 Hz light flicker did not enhance the microglia-dependent synapse pruning**

**a** Representative images of Iba1 immunostaining in three groups, scale bar: 100 μm. **b** Quantification of Iba1^+^ microglia density (WT: n = 5 mice, 16p: n = 5 mice, 16p+40Hz-14d: n = 5 mice). **c** Representative images of Iba1 and CD68 co-immunostaining in three groups, scale bar: 10 μm. **d - f** Quantification of microglia morphology including branch length per cell, branches per cell, and junctions per cell (WT: n = 20 fields from 5 mice, 16p: n = 20 fields from 5 mice, 16p+40Hz-14d: n = 20 fields from 5 mice). **g** Quantification of mean Iba1 intensity. **h** Quantification of CD68 volume ratio in microglia (WT: n = 56 cells from 5 mice, 16p: n = 46 cells from 5 mice, 16p+40Hz-14d: n = 44 cells from 5 mice). **i** Representative images of Iba1 and VGLUT1 co-immunostaining in three groups (WT: n = 32 cells from 5 mice, 16p: n = 37 cells from 5 mice, 16p+40Hz-14d: n = 43 cells from 5 mice), scale bar: 10 μm. **j** Quantification of VGLUT1 volume ratio in microglia. ns, not significant, *P < 0.05, **P < 0.01, ***P < 0.001, unpaired t test and Mann-Whitney U test.


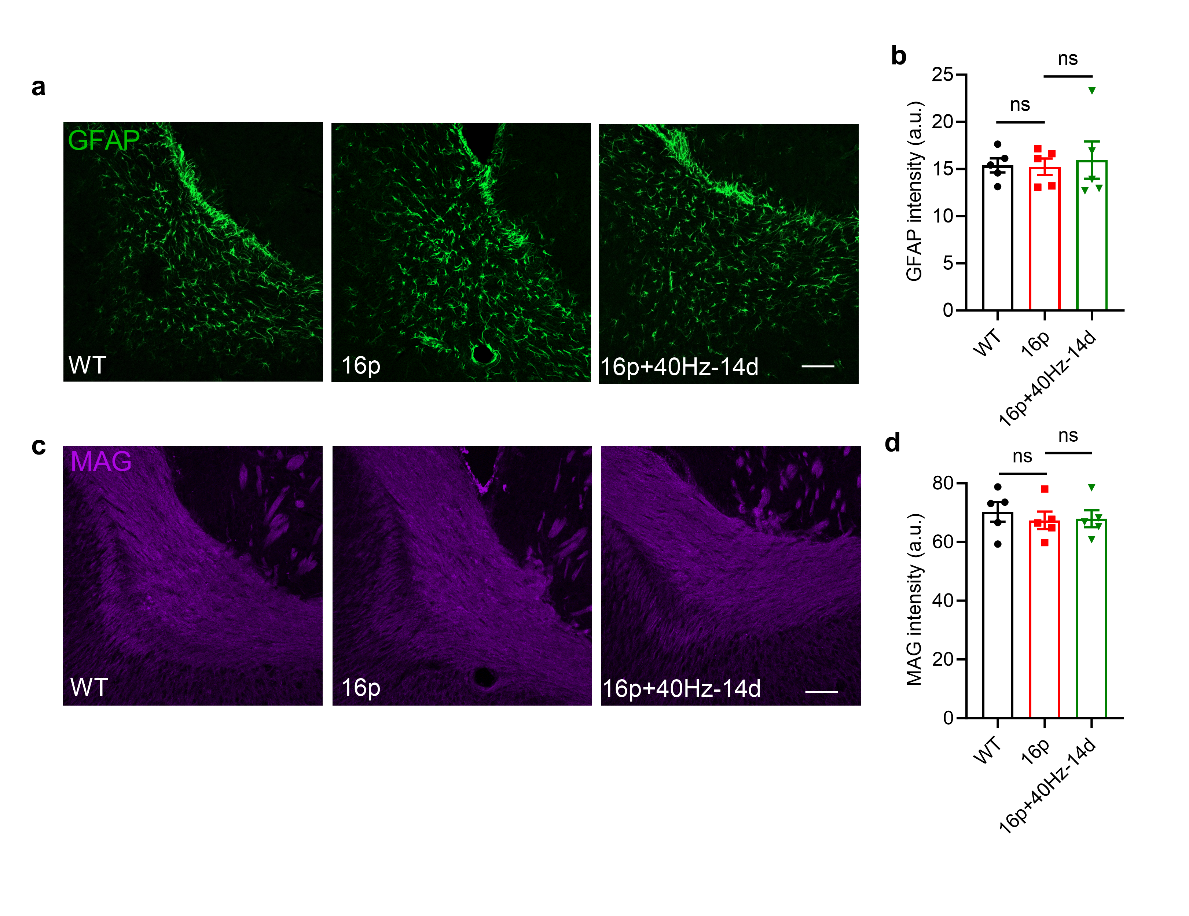


**Supplementary Fig. 9 40 Hz light flicker did not affect the astrogliosis and myelination**

**a** Representative image of astrocytes in the corpus callosum in three groups, scale bar: 100 μm. **b** Quantification of astrocytes intensity (WT: n = 5 mice, 16p: n = 5 mice, 16p+40Hz-14d: n = 5 mice). **c** Representative image of myelination in the corpus callosum in three groups, scale bar: 100 μm. **d** Quantification of myelination intensity (WT: n = 5 mice, 16p: n = 5 mice, 16p+40Hz-14d: n = 5 mice). ns, not significant, unpaired t test.


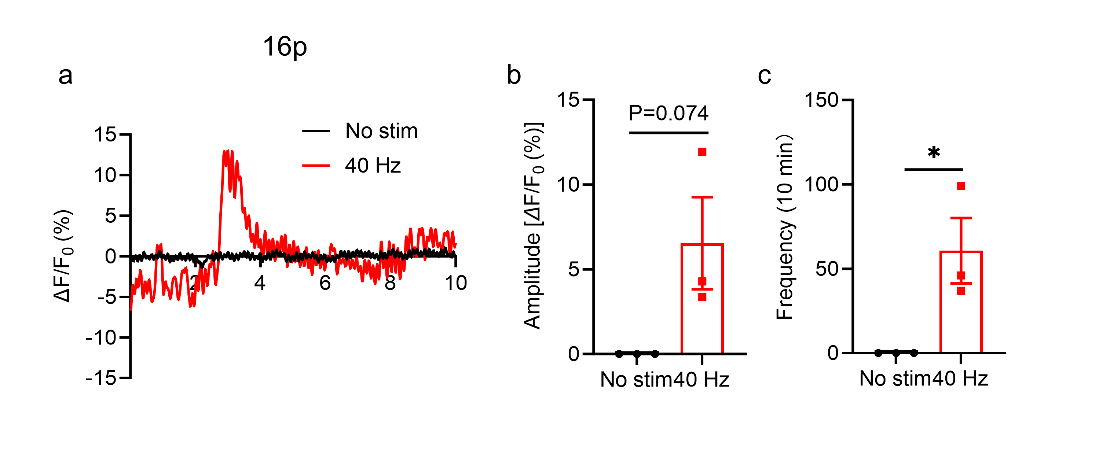


**Supplementary Fig. 10 40 Hz light flicker induced the adenosine release in 16p11.2 deletion female mice**

**a** Representative trace of adenosine signaling during optical fiber recording in 16p11.2 deletion female mice. **b** Quantification of the amplitude of adenosine signaling before (10 minutes) and during 40 Hz light flicker (10 minutes). **c** Quantification of the frequency of adenosine signaling before and during 40 Hz light flicker (n = 3 mice). *P < 0.05, unpaired t test.


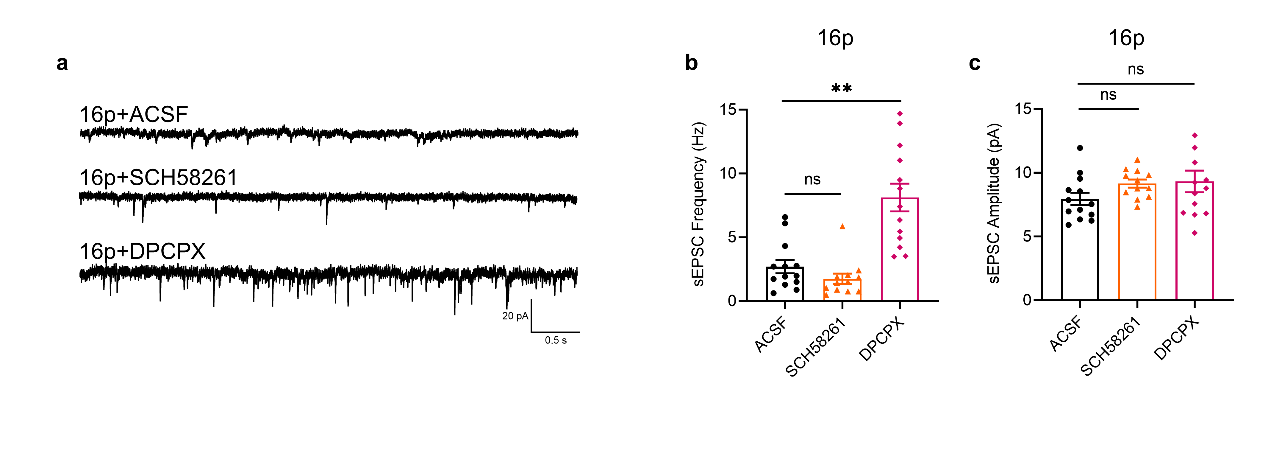


**Supplementary Fig. 11 A_1_ receptor antagonist increased the excitatory transmission in 16p11.2 deletion female mice**

**a** Representative traces of sEPSC in three groups, scale bar: 20 mV/0.5 s. **b** Quantification of sEPSC frequency. **c** Quantification of sEPSC amplitude (ACSF: n = 13 cells from 4 mice, SCH58261 (0.1 μM): n = 12 cells from 3 mice, DPCPX (0.3 μM): n = 13 cells from 3 mice). ns, not significant, **P < 0.01, one-way ANOVA with Tukey's *post hoc* test or Kruskal-Wallis test with Dunn's *post hoc* test.

**
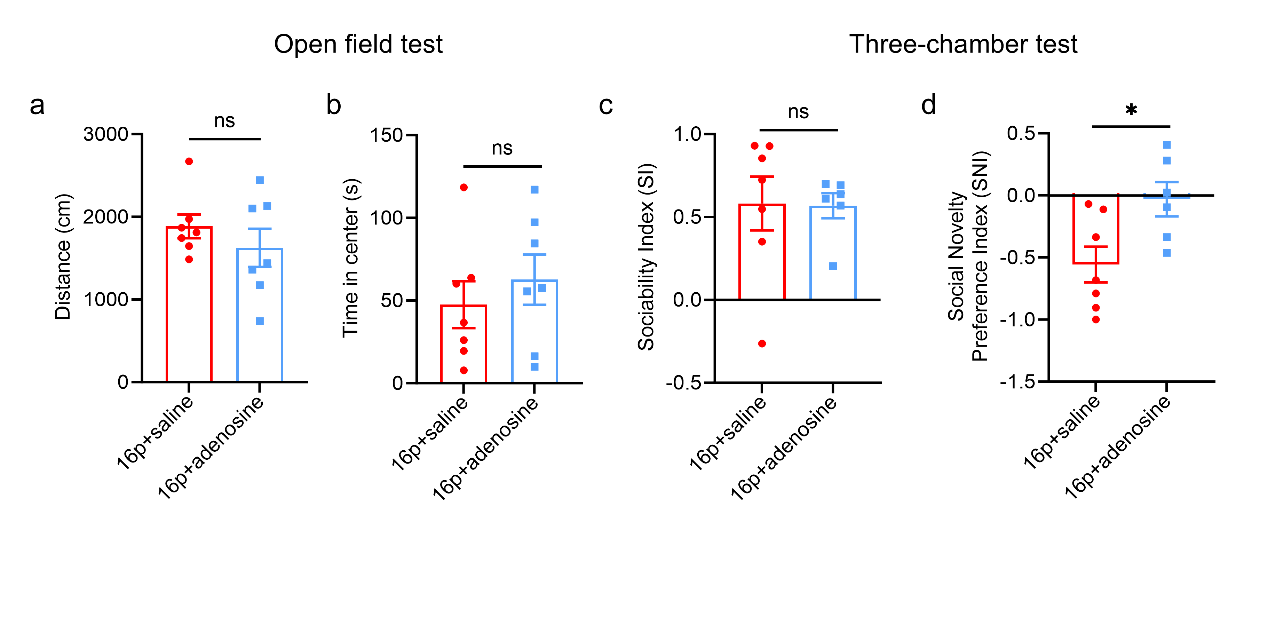
**

**Supplementary Fig. 12 Adenosine alleviated social novelty deficit in 16p11.2 deletion female mice *in vivo***

**a** The distance traveled in the open field, and **b** the time in the center in the open field test (16p+saline: n = 7 mice, 16p+adenosine: n = 7 mice). **c** Sociability index in the three-chamber test. **d** Social novelty preference index in three-chamber test (16p+saline: n = 7 mice, 16p+adenosine: n = 6 mice). ns, not significant, *P < 0.05, unpaired t test.
